# Consequences of Amyloid‐β Deficiency for the Liver

**DOI:** 10.1002/advs.202307734

**Published:** 2024-03-02

**Authors:** Gayane Hrachia Buniatian, Ute Schwinghammer, Roman Tremmel, Holger Cynis, Thomas S. Weiss, Ralf Weiskirchen, Volker M. Lauschke, Sonia Youhanna, Isbaal Ramos, Maria Valcarcel, Torgom Seferyan, Jens‐Ulrich Rahfeld, Vera Rieckmann, Kathrin Klein, Marine Buadze, Victoria Weber, Valentina Kolak, Rolf Gebhardt, Scott L. Friedman, Ulrike C. Müller, Matthias Schwab, Lusine Danielyan

**Affiliations:** ^1^ Department of Clinical Pharmacology University Hospital of Tuebingen Auf der Morgenstelle 8 72076 Tuebingen Germany; ^2^ Dr. Margarete Fischer‐Bosch Institute of Clinical Pharmacology Auerbachstr. 112 70376 Stuttgart Germany; ^3^ University of Tuebingen 72074 Tuebingen Germany; ^4^ Department of Drug Design and Target Validation Fraunhofer Institute for Cell Therapy and Immunology Weinbergweg 22 06120 Halle (Saale) Germany; ^5^ Junior Research Group, Immunomodulation in Pathophysiological Processes Faculty of Medicine Martin‐Luther‐University Halle‐Wittenberg Weinbergweg 22 06120 Halle (Saale) Germany; ^6^ Children's University Hospital (KUNO) University Hospital Regensburg Franz‐Josef‐Strauss‐Allee 11 93053 Regensburg Germany; ^7^ Institute of Molecular Pathobiochemistry Experimental Gene Therapy and Clinical Chemistry RWTH University Hospital Aachen Pauwelsstr. 30 52074 Aachen Germany; ^8^ Department of Physiology and Pharmacology Karolinska Institute Stockholm 171 77 Sweden; ^9^ Innovative Technologies in Biological Systems SL (INNOPROT) Bizkaia Derio 48160 Spain; ^10^ H. Buniatian Institute of Biochemistry National Academy of Sciences of the Republic of Armenia (NAS RA) 5/1 Paruir Sevak St. Yerevan 0014 Armenia; ^11^ Rudolf‐Schönheimer Institute of Biochemistry Faculty of Medicine University of Leipzig Johannisstraße 30 04103 Leipzig Germany; ^12^ Division of Liver Diseases Icahn School of Medicine at Mount Sinai 1425 Madison Ave New York NY 10029 USA; ^13^ Institute for Pharmacy and Molecular Biotechnology IPMB Department of Functional Genomics University of Heidelberg Im Neuenheimer Feld 364 69120 Heidelberg Germany; ^14^ Departments of Biochemistry and Clinical Pharmacology and Neuroscience Laboratory Yerevan State Medical University 2‐ Koryun St Yerevan 0025 Armenia; ^15^ Cluster of Excellence iFIT (EXC2180) “Image‐guided and Functionally Instructed Tumor Therapies” University of Tübingen 72076 Tübingen Germany

**Keywords:** 5xFAD, eNOS, neprilysin, presenilin, TGFβ, VEGF, β‐secretase 1

## Abstract

The hepatic content of amyloid beta (Aβ) decreases drastically in human and rodent cirrhosis highlighting the importance of understanding the consequences of Aβ deficiency in the liver. This is especially relevant in view of recent advances in anti‐Aβ therapies for Alzheimer's disease (AD). Here, it is shown that partial hepatic loss of Aβ in transgenic AD mice immunized with Aβ antibody 3D6 and its absence in amyloid precursor protein (APP) knockout mice (APP‐KO), as well as in human liver spheroids with APP knockdown upregulates classical hallmarks of fibrosis, smooth muscle alpha‐actin, and collagen type I. Aβ absence in APP‐KO and deficiency in immunized mice lead to strong activation of transforming growth factor‐β (TGFβ), alpha secretases, NOTCH pathway, inflammation, decreased permeability of liver sinusoids, and epithelial‐mesenchymal transition. Inversely, increased systemic and intrahepatic levels of Aβ42 in transgenic AD mice and neprilysin inhibitor LBQ657‐treated wild‐type mice protect the liver against carbon tetrachloride (CCl_4_)‐induced injury. Transcriptomic analysis of CCl_4_‐treated transgenic AD mouse livers uncovers the regulatory effects of Aβ42 on mitochondrial function, lipid metabolism, and its onco‐suppressive effects accompanied by reduced synthesis of extracellular matrix proteins. Combined, these data reveal Aβ as an indispensable regulator of cell–cell interactions in healthy liver and a powerful protector against liver fibrosis.

## Graphical abstract

Hepatic deficiency of Amyloid beta (Aβ) leads to liver fibrosis reflected by increased cleavage of NOTCH, activation of hepatic stellate cells (HSC), decreased permeability of endothelial cells (LSEC), and epithelial‐mesenchymal transition (EMT) of hepatocytes. High hepatic Aβ levels provide powerful protection against liver fibrosis by reversing the aforementioned processes.

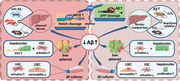

## 1 Introduction

Amyloid beta (Aβ) deposition in the brain is one of the main histopathological hallmarks of Alzheimer's disease (AD). To combat AD, various strategies directed at lowering cerebral Aβ by targeting Aβ itself or enzymes involved in amyloid precursor protein (APP) processing have been investigated in clinical trials.^[^ ^1^ ^]^ Among those, antibodies against different Aβ species such as oligomers, and fibrils in amyloid plaques^[^ ^2^ ^]^ are considered promising, with Aducanumab and Lecanemab as FDA‐approved antibodies to treat AD.^[^ ^3^ ^]^ The steady–state level of Aβ depends on the turnover of APP, a type1 transmembrane protein, which is processed via sequential cleavage by three proteases: *α*‐, *β*‐, and *γ*‐secretases.^[^ ^4^ ^]^ Cleavage of APP by β‐secretase (BACE), results in the generation of APP CTF‐99, from which Aβ is cleaved by presenilin 1 (PSEN1), the catalytic subunit of the gamma‐secretase complex.^[^ ^5^ ^]^

The liver is a key player in Aβ removal from the body, accounting for 60% of its clearance in the periphery.^[^ ^6^ ^]^ A high level of Aβ in the healthy liver is generated by the production of Aβ by liver cells, in addition to its delivery by blood.^[^ ^7^ ^]^ The functional role of Aβ in the liver remains to date unknown.

Because several key players of Aβ generation and degradation are involved in different pro‐fibrogenic pathways, we hypothesized that Aβ is essential for maintaining healthy liver function. For instance, PSEN1 is required for the cleavage and activation of NOTCH, which is characteristic of rodent and human fibrosis.^[^ ^8^, ^9^ ^]^ This together with our previous discovery of decreased APP and Aβ in human and rodent cirrhotic liver^[^ ^10^ ^]^ led us to the hypothesis that reduced APP and BACE1 may shift the γ‐secretase activity toward NOTCH cleavage thereby contributing to the loss of Aβ in fibrotic liver.

Another important crosslink between APP and NOTCH pathway is the dual activity of α‐secretases cleaving APP to non‐amyloidogenic APPα and NOTCH which is further processed by presenilin.

Aβ also decreases transforming growth factor‐β (TGFβ) in liver sinusoidal endothelial cells^[^ ^10^ ^]^ and reduces the activity of ubiquitin C‐terminal hydrolase L1 (UCHL1) in neuronal cells.^[^ ^11^ ^]^ UCHL1 is a deubiquitinase with profibrogenic effects in the liver that is strongly upregulated upon HSC activation and regulates their proliferation.^[^ ^12^ ^]^ These findings drove us to explore whether the loss of Aβ in APP knock‐out (APP‐KO) mice or its systemic decrease in anti‐Aβ immunized mice can lead to the development of fibrosis. Furthermore, we investigated the hepatoprotective effects of Aβ by inducing liver fibrosis with carbon tetrachloride (CCl_4_) in wild‐type (WT) mice with normal levels of Aβ and in transgenic AD mice (3xTg‐AD) with high systemic Aβ. To address the translational implications of maintaining high Aβ levels in the liver, we additionally tested the features of an Aβ‐degrading enzyme (neprilysin) inhibitor to protect against CCl_4_‐induced fibrosis. The engagement of Aβ in cell‐type specific functions of defense against liver fibrosis was investigated using a variety of primary cultures, cell lines, and human liver spheroids. This study provides the first direct evidence that Aβ protects against liver injury by targeting different key activators of hepatic fibrosis and determinants of liver sinusoidal permeability.

## 2 Results and Discussion

### 2.1 Aβ Regulates the Normal Function of Liver Endothelial Cells, Hepatocytes and Hepatic Stellate Cells

**Figure 1:**
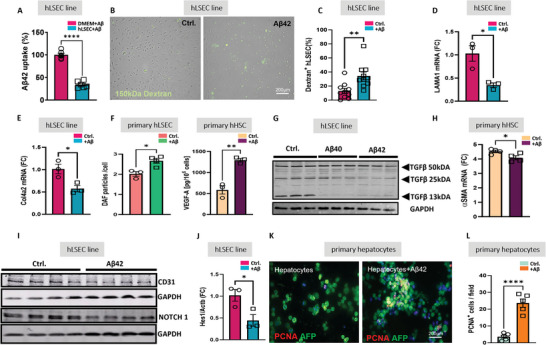
In vitro effects of Aβ42 on LSEC, HSC, and hepatocytes. A) Aβ42 utilization in human LSEC (hLSEC) line measured by a decrease of Aβ42 in cell culture supernatant normalized to culture medium without cells supplemented with Aβ42 (DMEM+ Aβ); B,C) live cell imaging and quantification of FITC‐Dextran 150 kDa uptake by hLSEC line incubated 24 h with and without Aβ42 (3000 pg mL^−1^, *n* = 10 per group); D) laminin 1(LAMA1) mRNA qPCR in hLSEC line (*n* = 3 per group); E) collagen 4a (Col4a2) mRNA qPCR in hLSEC line (*n* = 3 per group); F) Assessment of NO by Difluorofluorescein Diacetate (DAF) in primary hLSEC (*n* = 4 per group) and VEGF in primary human HSC (hHSC) by immunofluorescence staining (*n* = 3 per group); G) Western Blot (WB) analysis of TGFβ in hLSEC line incubated with Aβ40 or Aβ42 (1000 pg mL^−1^ each), GAPDH served as a loading control (*n* = 3 per group); H) αSMA mRNA qPCR in primary hHSC ±Aβ42 (*n* = 4 per group); I) WB of CD31 and NOTCH1 in hLSEC line +/‐ Aβ42 (*n* = 4 per group); J) Hes1 mRNA qPCR in hLSEC line ±Aβ42 (*n* = 3 per group); K) Immunofluorescence staining of PCNA in primary murine hepatocytes ±Aβ42 (1000 pg mL^−1^) counterstained with AFP and DAPI; L) Quantification of PCNA^+^ hepatocytes ±Aβ42 (1000 pg mL^−1^; *n* = 5 per group). The data are presented as means ± SEM. ^*^ *p* < 0.05, ^**^ *p* < 0.01, ^***^ *p* < 0.005, and ^****^ *p* < 0.001; two‐tailed Student's *t*‐test (A–F,H, J, L).

In line with previously shown Aβ uptake and degradation by hepatic stellate cells (HSC),^[^ ^10^ ^]^ here we demonstrate the utilization of Aβ by human liver sinusoidal endothelial cell (hLSEC) line (**Figure** **1**A) and by HepG2 cells (Figure S1A, Supporting Information). In hLSEC, the uptake of Aβ contributed to increased permeability reflected by intracellular accumulation of FITC‐dextran 150 kDa (Figure 1B,C), decreased production of collagen I (Col1a, Figure S1B, Supporting Information), laminin I and collagen IV (Figure 1D,E). As important components of the basement membrane, laminin, and collagen IV are acknowledged to raise the blood‐tissue barrier during liver fibrosis.^[^ ^13^ ^]^ The pore‐forming capacity of Aβ in brain capillaries leading to leakiness of the blood‐brain barrier (BBB) has been established in vivo and in culture.^[^ ^14^ ^]^ During cirrhosis and in culture conditions the initially differentiated LSEC lose fenestrations and acquire a de‐differentiated phenotype characterized by reduced capacity to produce nitric oxide (NO) and by increased expression of a marker of continuous endothelium CD31.^[^ ^15^ ^]^ Aβ induced NO generation in primary hLSEC and vascular endothelial growth factor (VEGF) release by primary human HSC (Figure 1F).

Furthermore, Aβ40 and 42 (1000 pg mL^−1^) reduced the proteolytic activation of TGFβ in a hLSEC line shown by decreased liberation of the 12.5–13 kDa monomer and the active 25 kDa fragment of TGFβ (Figure 1G) and downregulation of TGFβ mRNA (Figure S1B, Supporting Information). Notably, the effect of Aβ42 on TGFβ was more prominent than that of Aβ40 (Figure 1G). The above results suggest that Aβ may act as a potent mediator of paracrine signaling and crosstalk between hLSEC and HSC which is important for the transcellular exchange in liver sinusoids. Like primary murine HSCs,^[^ ^10^ ^]^ human primary HSCs responded to Aβ by reduced expression of smooth muscle alpha‐actin (αSMA) mRNA (Figure 1H). Thus, Aβ may govern the activation of HSC by the direct action on TGFβ expression in HSC and indirectly via a paracrine effect by decreasing its production by LSEC.

Along with CD31 as a marker of continuous endothelium, Aβ reduced another hallmark of fibrosis/cirrhosis, NOTCH, and its downstream effector Hes 1 (Figure 1I,J). While activated during liver fibrosis, the NOTCH pathway is inhibited in the brain affected by AD pathology.^[^ ^16^ ^]^ The down‐regulation of NOTCH 1 and Hes‐1 in hLSEC by Aβ shown here hints at the ability of Aβ to suppress the NOTCH‐cleaving activity of PSEN1. The activation of PSEN1 was observed in steatosis, inflammation, and liver fibrosis.^[^ ^17^ ^]^ Aβ also appears to downregulate the expression of TGFβ, NOTCH1, and alpha‐fetoprotein (AFP) in HepG2 cells (Figure S1C, Supporting Information). Taken together, Aβ suppresses multiple mechanisms commonly linked to fibrosis and hepatocellular carcinoma (HCC), such as the myofibroblastic transformation of HSC, upregulation of ECM proteins, activation of TGFβ, NOTCH signaling pathway and epithelial‐mesenchymal transition (EMT), which are all considered as harbingers of hepatocarcinogenesis.^[^ ^18^ ^]^

The unique regenerative capacity of hepatocytes in vivo is strongly limited in culture except when endogenous pathways promoting their growth in a healthy liver milieu are activated, such as Wnt/ β‐catenin.^[^ ^19^ ^]^ Aβ induced fivefold up‐regulation of proliferating cell nuclear antigen (PCNA) in primary murine hepatocytes (Figure 1K,L) reflecting the requirement for this peptide to repair and maintain functional liver cell mass, which is lost in chronic liver diseases.^[^ ^20^ ^]^

**Figure 2:**
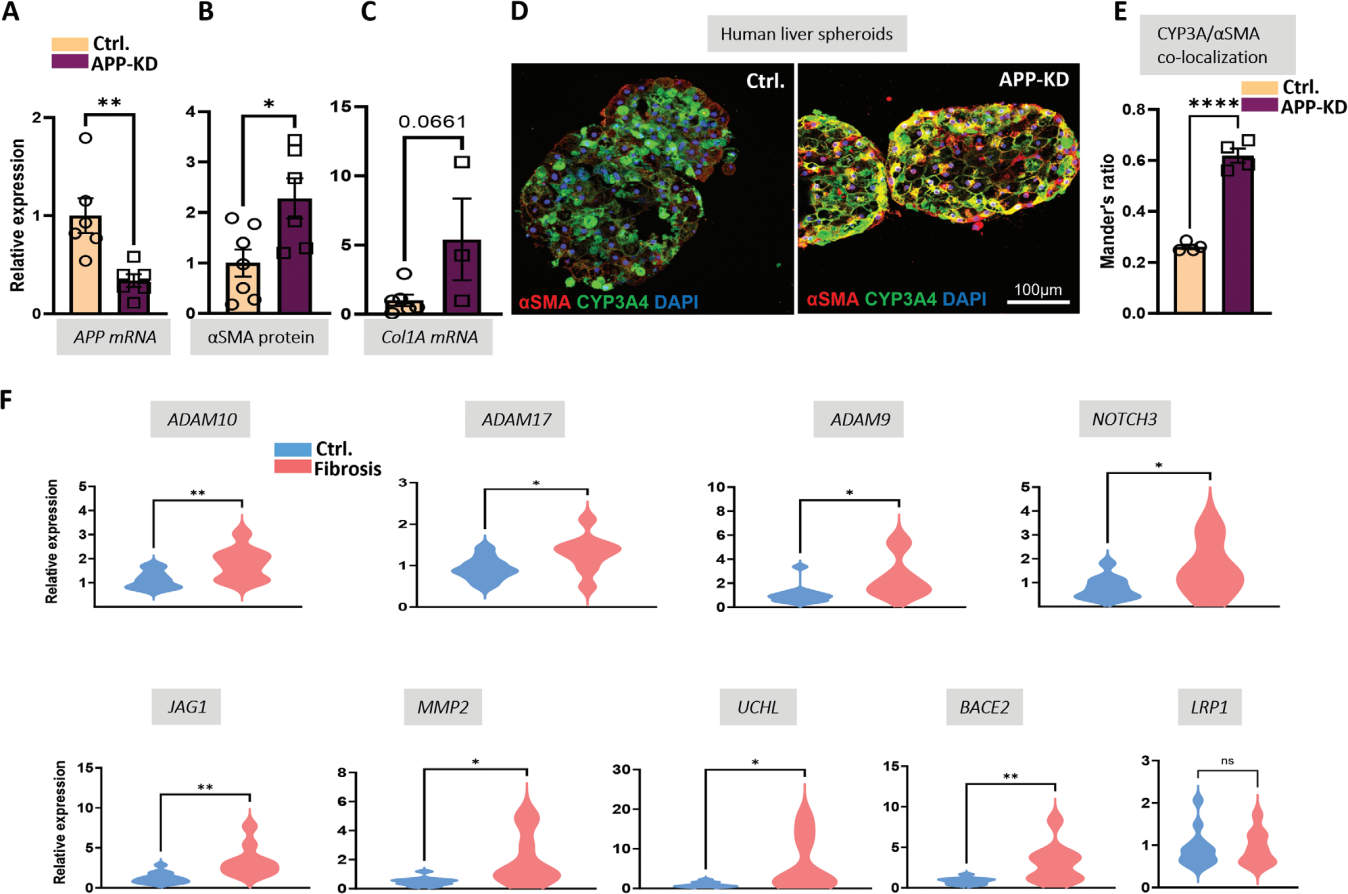
Phenotypic changes in APP knock‐down human liver spheroids and expression of APP processing enzymes and NOTCH activation genes in human fibrotic liver. A–C), Expression of APP, αSMA, and COL1A1 are shown in control (Ctrl.) and APP knock‐down (APP‐KD) human spheroids consisting of primary hepatocytes and HSC (*n*≥3); D) representative immunofluorescent images of CYP3A4 and αSMA in APP‐KD and control (Ctrl.) spheroids. Note that the overlap of αSMA and CYP3A4 drastically increases in APP‐KD, indicative of epithelial‐mesenchymal transition; E) quantification of the overlap of αSMA and CYP3A4 signals using Mander‘s Overlap Coefficient; F) qPCR of ADAM10, ADAM17, ADAM9, NOTCH3, JAG1, MMP2, UCHL, BACE2, and LRP‐1 mRNA in normal (Ctrl.) and fibrotic (fibrosis) human liver tissue. ^*^ *p* < 0.05, ^**^ *p* < 0.01, and ^****^ *p* < 0.001 using a two tailed *t*‐test (A–F), ns: not significant. For NOTCH3 and ADAM9 (in F) Mann–Whitney test was used.

Indeed, this dependency of survival and functionality of hepatocytes on the Aβ production was confirmed in 3D human liver spheroids containing primary human hepatocytes (PHH) and primary HSC. Knock‐down of APP (APP‐KD) in this organotypic model resulted in a significant downregulation of APP transcripts (**Figure** **2**A) leading to an upregulation of αSMA (*p* < 0.05) and a trend toward increased expression of COL1A1 (*p* = 0.067; Figure 2B,C). Furthermore, APP‐KD increased infiltration of αSMA+ HSCs and EMT of hepatocytes shown by the strong reduction of CYP3A4+ hepatocytes and the appearance of CYP3A4+ αSMA+ cells (Figure 2D,E).

### 2.2 Simultaneous Activation of NOTCH Signaling and Decreased BACE1 in Human Fibrotic Liver

Analysis of human fibrotic liver specimens provided the mechanistic background of the previously reported phenomenon of Aβ42 loss in rodents and human liver fibrosis.^[^ ^10^ ^]^ Upregulation of A disintegrin and a metalloprotease 9 (*ADAM9)* and alpha secretases *ADAM10* and *ADAM17* in fibrotic human livers (Figure 2F) implicates reduced APP processing along the amyloidogenic pathway. Remarkably, besides its α‐secretase activity for APP in non‐neural cells,^[^ ^21^ ^]^ ADAM9 promotes the production of bioactive TGFβ by cleaving the TGFβ latency‐associated peptide.^[^ ^22^ ^]^ Increased demand for NOTCH‐cleaving activity by γ‐secretase in human liver fibrosis is reflected by the upregulation of *NOTCH3* and *JAG1* mRNA (Figure 2F). Further on, upregulation of *MMP‐2* (gelatinase A) in fibrotic human tissue (Figure 2F), facilitates the degradation of Aβ40 and Aβ42.^[^ ^23^ ^]^ The next cause of Aβ loss in fibrosis is the up‐regulation of ubiquitin carboxyl‐terminal hydrolase L1 (UCHL1, Figure 2F) which is known to decrease the BACE catalyzed cleavage of an APP fragment, C99, hereby reducing the Aβ levels in vitro as shown previously in HUCH cells.^[^ ^24^ ^]^ Conversely, the upregulation of Aβ42 decreases the activity of UCHL1 via activation of NF‐κB pathway and BACE1.^[^ ^11^ ^]^ The antagonistic relationship of UCHL1 to Aβ and BACE explains the Aβ loss. The next Aβ reducing event in human fibrosis is the upregulation of *BACE2* (Figure 2F). This homolog of BACE1 cleaves wild‐type APP efficiently within the Aβ region hereby limiting the production of Aβ in BACE2‐expressing tissues.^[^ ^25^ ^]^

In addition to the impaired generation and increased degradation of Aβ, the hepatic content of Aβ may also be influenced by the trans‐ and intracellular transport of the peptide delivered through the bloodstream. Among the Aβ transporters, low‐density lipoprotein receptor‐related protein 1 (LRP‐1) is responsible for Aβ efflux from the brain and has been shown to significantly impact Aβ uptake by the liver.^[^ ^26^ ^]^ Our data show that at least at the RNA level LRP‐1 is not affected in pediatric patients with hepatic fibrosis (Figure 2F). However, acknowledging the existence of a great variety of other Aβ transporters and Aβ‐binding proteins in the blood,^[^ ^27^ ^]^ the extent to which changes in transporter systems contribute to the hepatic content of Aβ and the progression of fibrosis remains a subject for future investigations.

### 2.3 Passive Aβ Immunization and APP Knockout Lead to the Development of Liver Fibrosis

To explore the consequences of partial or complete loss of Aβ for liver function, we employed Aβ‐neutralizing passive immunization and APP‐KO models to deplete or reduce Aβ levels. For the partial loss of Aβ, mice with high and normal systemic levels of Aβ42, 5xFAD, and wild type C57Bl/6J (WT) respectively, were immunized with the mouse monoclonal antibody against the N terminus of Aβ42, 3D6. This antibody recognizes both soluble Aβ and insoluble Aβ in vivo and in vitro.^[^ ^28^, ^29^ ^]^ 3D6 treatment led to a significant intrahepatic decrease of Aβ42 in all immunized animals, starting from 6‐week immunization in WT to 6‐week and 8‐month immunization in 5xFAD mice (Figure S2A–C, Supporting Information).

The unchanged Aβ40 levels in response to immunization (Figure S2A–C, Supporting Information) are in line with previously reported ineffectiveness of 3D6 to lower Aβ40 in the brain of the Tg2576 mouse model of AD.^[^ ^29^ ^]^ Surface plasmon resonance spectroscopy confirmed that 3D6, which is mainly directed to the human Aβ, binds murine Aβ42 with a K_D_ of 185 nm (Figure S2D, Supporting Information). While in 3D6 immunized WT and 5xFAD mice the level of Aβ42 is partially retained, the genetic deletion of APP in APP‐KO mice, reflected by the absence of APP at the protein and mRNA level (Figure S3A, Supporting Information), is a dead‐end situation for the production of Aβ, since APP is the only source of Aβ42.

**Figure 3:**
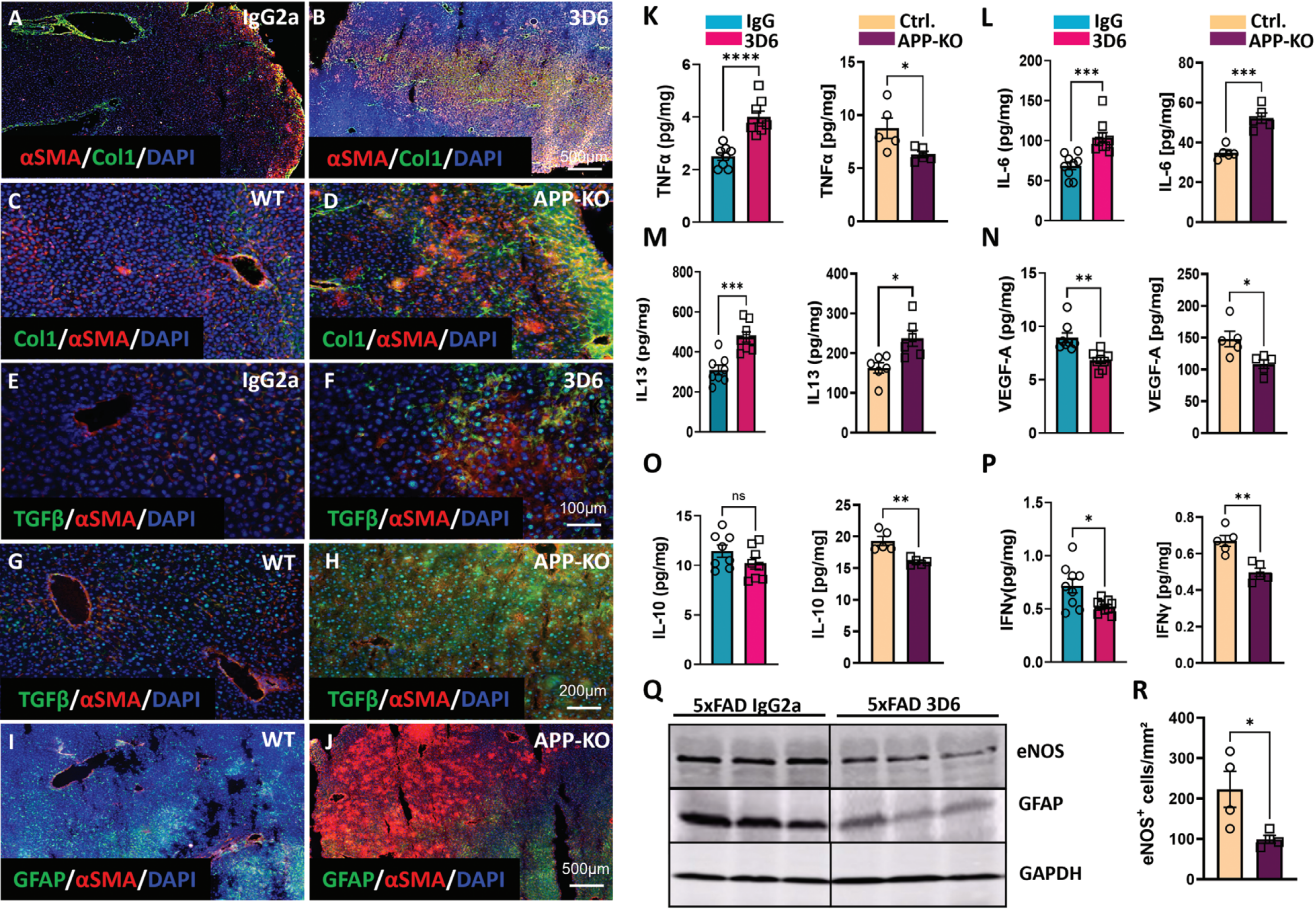
Assessment of fibrotic, inflammatory, and liver sinusoidal permeability markers in APP‐KO and 3D6‐immunized 5xFAD mice. A–J) Representative images of APP‐KO versus WT and 5xFAD mouse liver sections after 8‐month immunization with 3D6 versus IgG2a control antibodies. Immunofluorescence staining of (A–D) αSMA /Col1 /DAPI; E–H) TGFβ /αSMA /DAPI; I,J) GFAP /αSMA /DAPI (*n* = 4 per group); K–P) Multiplex analysis of TNFα, IL‐6, IL‐13, VEGF‐A, IL‐10, and IFNγ in liver homogenates of APP‐KO versus WT (*n* = 5 per group) and 5xFAD mice after 8‐month immunization with 3D6 versus IgG2a control antibodies (*n* = 9 per group); Q) WB of eNOS and GFAP in liver homogenates of 5xFAD mice after 8‐month immunization with 3D6 versus IgG2 control antibodies (*n* = 3 per group); R) Quantification of eNOS+ cells in liver sections of APP‐KO versus WT mice in 10 liver slices from of *n* = 3 mice per group. The data are presented as means ± SEM. ^*^ *p* < 0.05, ^**^ *p* < 0.01, ^***^ *p* < 0.005, and ^****^ *p* < 0.001; two‐tailed Student's *t*‐test (K–P, R).

Partial loss of bioactive Aβ in immunized wild type and 5xFAD mice (i‐WT and i‐5xFAD) and the complete loss of Aβ in APP‐KO mice resulted in the widely accepted signature of fibrosis illustrated by increased production of interstitial Col1, αSMA, and TGFβ (**Figure** **3**A–H; Figure S3B–D, Supporting Information), laminin positive microvessels and Col4 (Figure S3C,D, Supporting Information). In APP‐KO livers, advanced fibrosis/cirrhosis is evident by structural heterogeneity of liver tissue, large areas enriched with Col1 and inhabited by HSC strongly expressing αSMA, while largely lacking glial fibrillary acidic protein (GFAP, Figure 3I,J). Of note, the areas with αSMA‐positivity were clearly delineated from GFAP+ areas (Figure 3J) representing the non‐myofibroblastic phenotype of HSC.^[^ ^30^ ^]^ The changes in fibrotic, inflammatory, and endothelial permeability markers were evidenced by upregulation of αSMA, Col1, and TNFα in 6‐week immunization in i‐WT (Figure S4, Supporting Information) and additionally by IL‐6, and IL‐13 in 6‐week i‐5xFAD (Figure S5, Supporting Information) which remained elevated in 8‐month immunized animals (Figure 3K–M). Reduced level of TNFα in APP‐KO (Figure 3K) most likely reflects the sensitivity of this cytokine to Aβ levels and its engagement in Aβ production as shown for astroglia and neurons.^[^ ^31^ ^]^ Our data hint at possible Aβ associated TNFα activity in the liver that intensifies the amyloidogenic processing of APP, which cannot be realized in APP‐KO. Besides deep morphological restructuring of the liver tissue, strong αSMA, Col1, TGFβ expression, and inflammatory response reflected by IL‐6 and IL‐13, a higher degree of fibrosis in APP‐KO livers, compared to i‐5xFAD, is demonstrated by additionally decreased levels of IL‐10 and IFNγ (Figure 3O,P). Both cytokines prevent chronic fibroproliferative diseases by inhibiting TGFβ.^[^ ^32^ ^]^ IFNγ also inhibits HSC activation^[^ ^33^ ^]^ and like VEGF contributes to the permeability of liver sinusoids.^[^ ^34^, ^35^ ^]^

The impaired permeability of liver sinusoids in i‐5xFAD, i‐WT, and APP‐KO mice was also evidenced by decreased production of VEGF (Figure 3N; Figure S5B, Supporting Information) and eNOS (Figure 3Q–R) and by abruptly decreased expression of GFAP (Figure 3I,J), which is associated with decreased barrier features of blood–liver and blood–brain interfaces.^[^ ^36^ ^]^ The synergistic activities and mutual inducibility of GFAP, Aβ, and NO have previously been demonstrated in mouse primary astrocytes.^[^ ^37^ ^]^

Unlike the liver in which Aβ level correlates with eNOS that supports healthy organ function, in the brain some studies attribute the tissue damage and cognitive impairment to a deficit of eNOS,^[^ ^38^ ^]^ whereas others, find a link between upregulation of eNOS and increased deposits of Aβ during AD.^[^ ^39^ ^]^ In the brain of APP‐KO mice, suppressed hippocampal expression of eNOS was paralleled by upregulation of TGFβ (Figure S6A, Supporting Information). Opposite to its profibrogenic function in the liver, the brain TGFβ is in demand for the development of neurons.^[^ ^40^ ^]^ Moreover, impairment of TGFβ signaling leads to exacerbated deposition of Aβ.^[^ ^41^ ^]^ Also, the upregulation of laminin in the brains of APP‐KO and Col IV in i‐5xFAD (Figure S6A–C, Supporting Information), supports the physiological priorities of CNS to maintain the barrier function of the brain capillaries.^[^ ^42^ ^]^ Contrary to the BBB in a healthy brain, the establishment of the blood‐liver barrier by extracellular matrix (ECM) proteins (Figures S3C,D and S6D, Supporting Information) followed by displacement of permeability markers, VEGF, IFNγ, and eNOS (Figure 3N,P–R) leads to the progression of liver fibrosis.

### 2.4 Aβ Reduction or Complete Loss is Linked to NOTCH Pathway Activation

**Figure 4:**
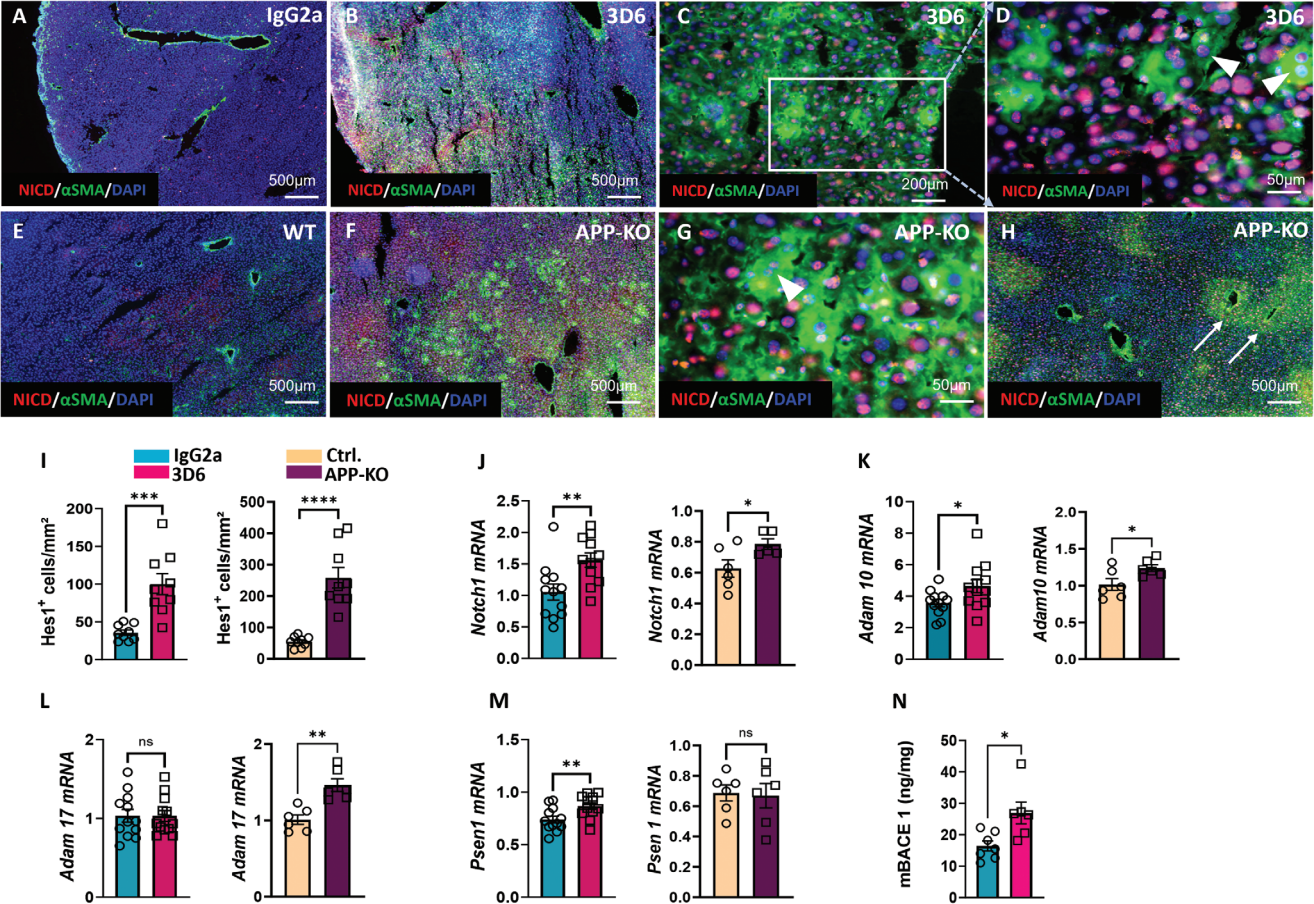
NOTCH pathway in APP‐KO and 3D6‐immunized 5xFAD mice. A–H) Representative images of APP‐KO versus WT and 5xFAD mouse livers after 8‐month immunization with 3D6 versus IgG2a control antibodies. Immunofluorescence staining of liver sections for NICD/αSMA/DAPI, (*n* = 4 per group); Arrowheads in G and D indicate αSMA/NICD positive binucleated cells putatively reflecting the ongoing EMT in hepatocytes; Arrows in H indicate microvessels surrounded by multiple layers of αSMA+/NICD+ HSC; I) Quantification of Hes1+ cells in liver sections of APP‐KO versus WT and 3D6 versus IgG2a treated 5xFAD mice from, *n* = 9 per group. J–M) qPCR of *Notch 1, Adam‐10, Adam‐17*, and *Psen1* in liver samples of APP‐KO versus WT (*n* = 12 per group) and 5xFAD mice after 8‐month immunization with 3D6 versus IgG2a control antibodies (*n* = 6 per group); N) BACE1 protein assessed by ELISA in liver homogenates of 5xFAD mice after 8‐month immunization with 3D6 versus IgG2a control antibodies (*n* = 7 per group). The data are presented as means ± SEM. ^*^ *p* < 0.05, ^**^ *p* < 0.01, and ^****^ *p* < 0.001; two‐tailed Student's *t*‐test (I–N).

In human and rodent fibrosis TGFβ‐signaling is accompanied by simultaneous activation of its partner pathway NOTCH.^[^ ^8^, ^43^ ^]^ The activation of NOTCH depends on its cleavage by alpha‐secretases ADAM10 and ADAM 17 at site S2 and gamma‐secretase‐mediated (S3 cleavage) release of NOTCH intracellular domain (NICD) into the cytoplasm. NICD further translocates into the nucleus, where it activates transcription of its target genes including Hes1.^[^ ^44^ ^]^ Immunization of 5xFAD mice and APP‐KO resulted in large areas populated with αSMA positive and negative cells with nuclear localization of NICD (**Figure** **4**A–H). These areas also contained microvessels surrounded by multiple layers of αSMA^+^/NICD^+^ HSC (Figure 4H).

The synergism between NOTCH and TGFβ is conditioned by direct interactions between NICD and an intracellular transducer of TGFβ signals *Smad3*, resulting in Hes1 expression.^[^ ^45^ ^]^ Indeed, in i‐5xFAD and APP‐KO mice NOTCH‐TGFβ interactions are evidenced by the overall increase in Hes1+ cells (Figure 4I) and co‐occurrence of Hes1 in areas populated by αSMA‐positive HSC (Figure S3C,D, Supporting Information).

Another event common to fibrosis and carcinogenesis requiring the TGFβ/NOTCH pathways’ synergism is EMT.^[^ ^46^, ^47^ ^]^ Notably, in areas frequently occurring in i‐5xFAD and APP‐KO livers, NICD appeared in binuclear hepatocyte‐like cells strongly expressing αSMA (Figure 4C,D,G). Similar to our results on APP‐KD in human liver spheroids, showing an ongoing EMT in hepatocytes (Figure 2D,E), in i‐5xFAD, and in APP‐KO mice αSMA/NICD positive binucleated cells are likely to reflect ongoing EMT in hepatocytes (arrowheads in Figure 4D,G).

Up‐regulation of *Notch1* and *Adam10* in i‐5xFAD and APP‐KO livers (Figure 4J,K) indicates intense S2 cleavage and maturation of the NOTCH receptor.^[^ ^48^ ^]^ Additional upregulation of ADAM17 was seen only in APP‐KO mice (Figure 4L) further supporting the notion of a higher degree of NOTCH activation in mice completely lacking the Aβ production. The propagation of NOTCH and TGFβ signaling in APP‐KO and i‐5xFAD livers is further confirmed by strong staining of HSC for Hes1 and αSMA (Figure S3C,D, Supporting Information), the common targets of TGFβ and NOTCH signaling.^[^ ^49^ ^]^

In neural cells, increased TNFα leads to elevated β‐secretase, Aβ,^[^ ^31^ ^]^ and Aβ‐associated γ‐secretase activity,^[^ ^50^ ^]^ while the genetic deletion of TNFα in 5xFAD mice attenuates cerebral Aβ generation via reduction of functionally active PSEN1 and BACE1.^[^ ^51^ ^]^ Thus, enhanced production of TNFα (Figure 3K), Psen1, and BACE1 (Figure 4M,N) in i‐5xFAD livers, seems to reflect an attempt to re‐establish Aβ homeostasis as a defense mechanism against 3D6 caused loss of Aβ.

### 2.5 High Systemic and Intrahepatic Levels of Aβ in 3xTg‐AD Mice Protect from Carbon Tetrachloride‐Induced Liver Fibrosis

**Figure 5:**
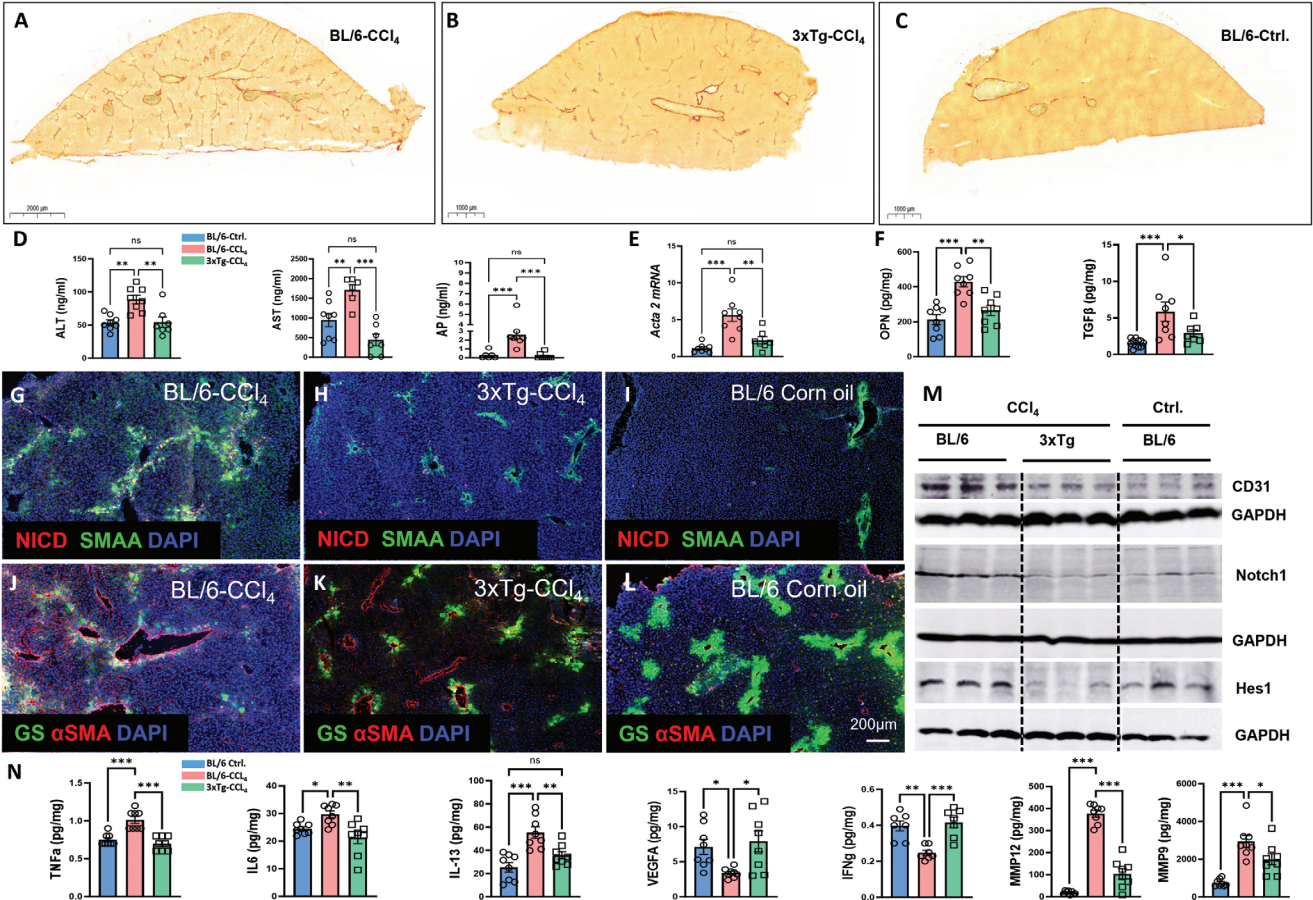
Aβ protects 3xTg‐AD mice from CCl_4_‐induced fibrosis. Data sets acquired from liver samples of CCl_4_‐ treated 3xTg‐AD (3xTg‐CCl_4_) versus BL/6 (BL/6‐CCl_4_) and Corn oil treated BL/6 controls (BL/6‐Ctrl.) after 5 weeks of CCl_4_ versus corn oil treatment; A–C) Representative images of Sirius Red staining (*n* = 4 per group); D) plasma liver enzymes (AST, ALT, and AP), *n* = 8 per group; E) qPCR of αSMA (acta2) mRNA (*n* = 8 per group); F) Multiplex Analysis of Osteopontin (OPN) and TGFβ (*n* = 8 per group); G–L) Immunofluorescence staining of liver sections for (G–I) NICD/ αSMA/DAPI, (*n* = 4 per group); J–L) glutamine synthetase (GS)/ αSMA/ DAPI, (*n* = 4 per group); M) Western Blot of CD31, Notch1, Hes1 (*n* = 3 per group); N) TNFα, IL‐6, IL‐13, VEGF‐A, IFNγ, MMP‐12, and MMP‐9 multiplex analysis of liver homogenates (*n* = 8 per group). The data are presented as means ± SEM. ^*^ *p* < 0.05, ^**^ *p* < 0.01, ^***^ *p* < 0.005, and ^****^ *p* < 0.001; One‐way ANOVA with Bonferroni”s post hoc test (D–F, N) and Kruskal–Wallis for AP analysis (in D).

To explore putative protective features of Aβ against liver fibrosis in vivo, we assessed fibrosis in WT (BL/6) and 3xTg‐AD mice harboring human APP /PSEN1 and tau mutations^[^ ^52^ ^]^ after 5‐week CCl_4_ treatment. In comparison to 3xTg‐AD mice (3xTg‐CCl_4_), serum and intrahepatic levels of human and murine Aβ (Figure S7, Supporting Information) were decreased in CCl_4_‐treated WT mice (BL/6‐CCl_4_). This decrease was strongly counterbalanced by the overproduction of Aβ in transgenic 3xTg‐AD mice (3xTg‐CCl_4_). According to our in vitro studies showing the effect of Aβ on HSC, LSEC, and hepatocytes, livers of 3xTg‐AD with high Aβ levels should better withstand profibrotic influences of CCl_4_ compared to WT. Indeed, in 3xTg‐CCl_4_, the profibrotic markers, i.e., collagen (**Figure** **5**A–C), liver enzymes (Figure 5D), αSMA (Figure 5E), osteopontin (OPN) and TGFβ (Figure 5F), along with the key components of the NOTCH pathway, NICD, Notch1 and Hes1 (Figure 5G–I,M) were all suppressed compared to CCl_4_‐BL/6 in which serum and liver Aβ level were 50% lower (Figure S7, Supporting Information).

In a healthy liver glutamine synthetase (GS) an enzyme converting glutamate and ammonia into glutamine is strongly expressed in specialized pericentral hepatocytes arranged in 2–3 rows around the central venules (Figure 5L), a zone that has been found to be sensitive to CCl_4_ toxicity.^[^ ^53^ ^]^ Accordingly, in BL/6‐CCl_4_ livers GS expression was nearly absent from the pericentral hepatocytes (Figure 5J) while in 3xTg‐CCl_4_ livers (Figure 5K) an energy‐consuming production of glutamine by perivenous cells was still maintained, however it was reduced to one layer in comparison to the oil‐treated BL/6 control (Figure 5L). Consistent with the notion that TGFβ induces EMT in hepatocytes, hallmarked by a decrease in E‐cadherin (E‐Cad) expression and acquisition of myofibroblastic markers,^[^ ^54^ ^]^ reduced E‐Cad and appearance of E‐Cad/αSMA positive cells were present only in BL/6‐CCl_4_ mouse liver in contrast to a higher expression and clear αSMA‐negativity of E‐Cad in pericentral hepatocytes in 3xTg‐CCl_4_ and in the vehicle (corn oil) treated BL/6 (Figure S8A–C, Supporting Information).

Oxidative stress is a well‐recognized precipitant of liver injury during fibrosis. There is a decrease in antioxidant superoxide dismutases including SOD1 in several models of rodent fibrosis including CCl_4._^[^ ^55^ ^]^ Consistent with other hepatoprotective features of Aβ, the 3xTg‐CCl_4_ group displayed increased SOD1 and downregulated DNA oxidation products 8‐OH‐dG (Figure S8D–F, Supporting Information). In line with data from APP‐KO and i‐5xFAD models, high level of Aβ in 3xTg‐CCl_4_ led to decreased CD31 (Figure 5M), TNFα, IL‐6, IL‐13, and elevated IFNγ and VEGF (Figure 5N). Because metalloproteinase 9 (MMP‐9) is known for its pronounced profibrotic and Aβ‐degrading activities,^[^ ^56^ ^]^ the drastically decreased levels of MMP‐9 in CCl_4_‐3xTg versus BL/6‐CCl4 (Figure 5N) represent a plausible explanation for very mild fibrosis in 3xTg animals with Aβ‐excess and constantly elevated intrahepatic levels of Aβ. Also, MMP‐12 which can control liver inflammation and IL‐13‐induced fibrosis^[^ ^57^ ^]^ was downregulated in 3xTg‐CCl_4_ mice along with IL‐13 decrease (Figure 5N).

**Figure 6:**
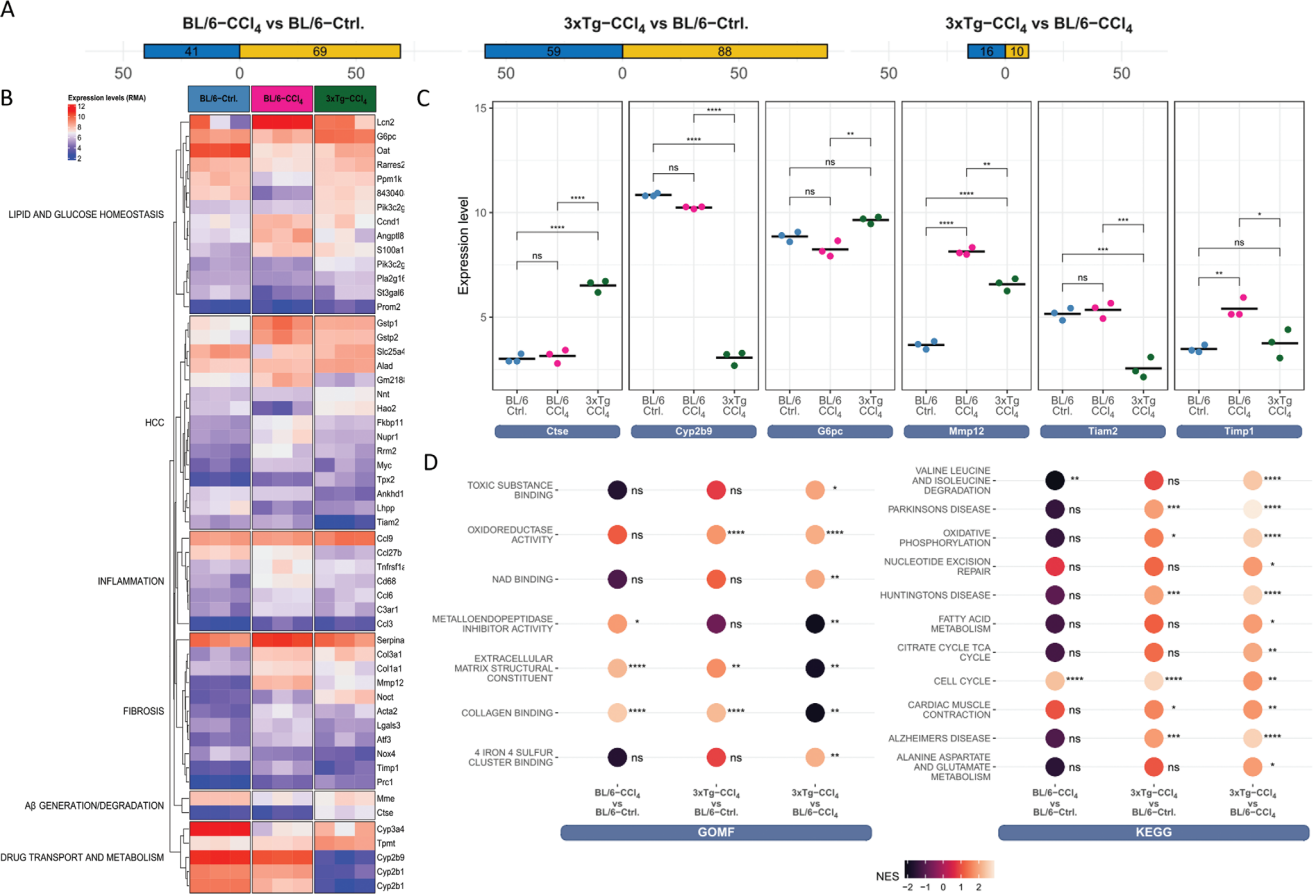
Transcriptome analysis of CCl_4_‐treated BL/6 and 3xTg‐AD mouse livers. A–C) Liver samples from CCl_4_‐treated BL/6 and 3xTg and corn oil‐treated BL/6 control mice were analyzed using Clariom S arrays. A) Number of differentially expressed genes (DEGs) using RMA and limma analysis. Results were filtered for FDR p‐value <0.05 and absolute logFC >1.5. B) Heatmap of selected DEGs categorized into lipid and glucose homeostasis, hepatocellular carcinoma (HCC), inflammation, fibrosis, Aβ generation and degradation, and drug transport and metabolism; C) Microarray gene expression of fibrosis markers; D) Enriched Gene Ontology (GO) molecular function and KEGG pathways. The color legend indicates the degree of normalized enrichment score (NES). Significance is reflected by *p* ^****^ = 10^−4^, ^***^ = 10^−3^, ^**^10^−2^, ^*^ = 0.05, and ns = not significant.

To gain insights into signaling pathways involved in hepatoprotective effects of Aβ excess in 3xTg‐AD mice, the liver transcriptome of solvent (corn oil)‐ and CCl_4_‐treated WT (BL/6) was compared to that of CCl_4_‐treated 3xTg‐AD mice (*n* = 3 per group). A hierarchical cluster analysis of the transcriptomic data showed clustering of each group of three mice (Figure S9A, Supporting Information). Compared to the corn oil control (BL/6‐ctrl), 110 genes were strongly deregulated in BL/6‐CCl_4_ (**Figure** **6**A). As expected, in BL/6 mice CCl_4_ treatment affected inflammation, fibrogenesis, oncogenesis markers (HCC), and genes involved in lipid and glucose metabolism (Figure 6B).

Out of 26 differentially expressed genes (DEGs) in 3xTg‐CCl_4_ mice in comparison to BL/6‐CCl_4_ (Figure 6A), the Cyp2b family genes (Cyp2b9, Cyp2b10, and Cyp2b13) were among those exclusively downregulated in 3xTg‐AD‐CCl_4_ mice (Figure 6B,C). Irrespective of the notion that these changes are likely to reflect a strain effect rather than CCl_4_‐treatment, this subfamily of genes being sex‐biased contributes to the promotion of fibrosis in female mice. A study with Cyp2b‐null mice reported resistance to diet‐induced steatotic disease in females provided by Cyp2b9, 10, and 13 deficiencies, while in males it was associated with higher susceptibility to nonalcoholic fatty liver disease (NAFLD).^[^ ^58^ ^]^ Across the preselected genes involved in fibrotic ECM remodeling, tissue inhibitor matrix metalloproteinase 1 (Timp1), and Mmp12 were significantly decreased in 3xTg‐CCl_4_ (Figure 6B,C).

Among carcinogenesis markers, an onco‐suppressor Hao2 which is decreased in HCC^[^ ^59^ ^]^ was upregulated, while the HCC‐ and EMT‐promoting marker, T‐cell lymphoma invasion and metastasis 2 gene (TIAM2), was prominently downregulated in 3xTgAD‐CCl_4_ (Figure 6B,C). The notion of the onco‐suppressive effect of high Aβ levels in the liver of 3xTg‐AD mice is further supported by decreased expression of lipocalin 2 (Lcn2), adipokine that plays a prominent role in lipogenesis and at the same time a reliable marker of poor prognosis of HCC. Lcn2 is upregulated in nonalcoholic steatohepatitis (NASH), NAFLD, and liver cirrhosis and in mice upon CCl_4_ injury as an indicator of liver damage.^[^ ^60^ ^]^ DEGs in 3xTg‐CCl_4_ involved in Aβ production and metabolism were represented by MME encoding Aβ‐degrading enzyme neprilysin and ctse encoding cathepsin E. Cathepsin E has been recently shown to regulate BACE‐1 expression and induce BACE1‐mediated production of Aβ in the brain.^[^ ^61^ ^]^ While MME expression was nearly equal in BL/6‐CCl_4_ and 3xTg‐CCl_4_, the increase in ctse in 3xTg‐CCl_4_ may additionally hint at the higher capacity of liver cells to generate Aβ in these mice. Strikingly, 3xTg‐CCl_4_ livers displayed an increased glucose‐6‐phosphatase α (g6pc) expression in comparison to BL/6‐CCl_4_ (Figure 6B,C). G6pc is the rate‐limiting enzyme of gluconeogenesis, the deficiency of which is the primary cause of glycogen storage disease type Ia (GSDI) in humans. GSDI is characterized by hypoglycemia, hepatic glycogen accumulation, and lipogenesis leading to steatosis and cirrhosis, tumorigenesis, and impaired oxidative phosphorylation (OxPhos) in the liver.^[^ ^62^ ^]^ In this sense, the G6pc serves as a crossing point of several pathways including lipogenesis, fatty acid metabolism, OxPhos, and β‐oxidation which all are differentially regulated in 3xTg‐CCl_4_ livers compared to BL/6‐CCl_4_ (Figure 6C; Figure S9B, Supporting Information).

According to GO and KEGG analysis (Figure 6C; Figure S9B, Supporting Information), pathways strongly associated with human and rodent liver fibrosis, such as extracellular matrix structural constituents, collagen binding organization and metabolism, metallopeptidase activity, cytokine‐mediated signaling, TNF superfamily cytokines, TGFβ activation, fatty acid metabolism, and apoptotic cell clearance were all downregulated in 3xTg‐CCl_4_ group in comparison to BL/6‐CCl_4_ (Figure 6C; Figure S9B, Supporting Information). This is also in line with results presented in Figure 5 showing significant changes in the expression of key components and/or regulators of some of these pathways (TNFα, TGFβ, Col1, MMP12, and 9) at the protein level. Importantly, the appearance of Alzheimer's, Parkinson's (PD), and Huntington's disease (HD) among top KEGG pathways differentially regulated in 3xTg‐CCl_4_ accounted primarily for the strong abundance of key genes of OxPhos (several subunits of complex I, III and IV) in all three disease pathways. In 3xTg‐CCl_4_, OxPhos was significantly upregulated in comparison to both BL/6‐CCl_4_ and BL/6‐corn oil controls (Figure 6D). Apparently, in contrast to the brain, where downregulated OxPhos is the common characteristic of AD, PD, and HD pathology, in humans^[^ ^63^ ^]^ and in 3xTg‐AD mice,^[^ ^64^ ^]^ the livers of 3xTg‐AD mice have high OxPhos activity which is in demand for normal liver function and it is impaired across fibrosis‐associated liver diseases.^[^ ^65^ ^]^ Moreover, findings by Santacatterina et al. have shown that the liver with reduced OxPhos is prone to the development of cancer.^[^ ^66^ ^]^

Overall, the transcriptome of 3xTg‐CCl_4_ favored a strong representation of genes involved in lipid metabolism, enhanced OxPhos and β‐oxidation, and downregulation of ECM components, inflammasome, fibrogenic, and HCC markers.

### 2.6 Inhibition of Aβ Degradation Prevents CCl_4_‐Induced Fibrosis

The dual‐action drug LCZ696 (brand name Entresto), comprising the neprilysin (NEP) inhibitor sacubitril and the angiotensin receptor antagonist valsartan has been safely used for more than a decade in the therapy of heart failure. LBQ657 which is the active metabolite of sacubitril has been previously reported to decrease TGFβ‐induced cardiac fibrosis.^[^ ^67^ ^]^ Recently, the administration of sacubitril/valsartan or the knockout of NEP ameliorated CCl_4_‐induced liver fibrosis in mice.^[^ ^68^ ^]^ These effects were however ascribed to the combined action of sacubitril/valsartan to diminish the profibrotic effects of angiotensin II and NPY receptor in the liver. Our previous in vitro results demonstrated a downregulation of TGFβ and αSMA in HSC exposed to LBQ657. However, this action of LBQ657 was completely dependent on the presence of Aβ42.^[^ ^10^ ^]^ Here, we sought to explore whether the sole inhibition of neprilysin by LBQ657 may slow down the progression of CCl_4_‐induced fibrosis in BL/6 mice.

**Figure 7:**
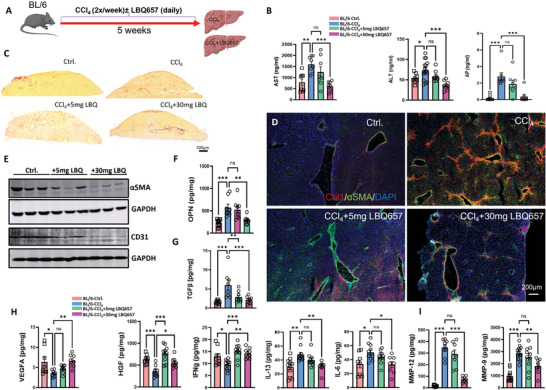
Inhibition of neprilysin protects BL/6 mice from CCl_4_‐induced fibrosis. A) Schematic presentation of treatment timeline: 5‐week CCl_4_‐ treatment (2x/week) of BL/6 mice ± two different dosages of neprilysin inhibitor sacubitrilat (LBQ657), 5 mg or 30 mg kg^−1^ body weight versus corn oil‐treated BL/6 controls (BL/6‐Ctrl.); B) plasma liver enzymes (AST, ALT, and AP), *n* = 8 per group; C) Sirius Red staining (*n* = 4 per group); D) Immunofluorescence staining of liver sections for Col1/ αSMA/DAPI, (*n* = 4 per group); E) Western Blot of αSMA and CD31 (*n* = 3 per group); F–I) Multiplex analysis of Osteopontin (OPN), TGFβ, TNFα, IL‐6, IL‐13, VEGF‐A, IFNγ, MMP‐12, and MMP‐9 in liver homogenates (*n* = 8 per group); The data are presented as means ± SEM. ^*^ *p* < 0.05, ^**^ *p* < 0.01, ^***^ *p* < 0.005, and ^****^ *p* < 0.001; One‐way ANOVA with Bonferroni‘s post hoc test (B, F–I) and Kruskal–Wallis test for AP analysis (in B).

Chronic administration of LBQ657 over a period of 5‐week CCl_4_‐treatment (**Figure** **7**A) ameliorated the entire set of fibrotic markers including liver enzymes, collagen, αSMA, OPN, TGFβ (Figure 7B–G), and inflammatory markers (Figure 7H). Similar to the effect of increased systemic/intrahepatic level of Aβ in 3xTg‐AD mice, inhibition of Aβ degradation by LBQ657 enhanced the hepatocyte growth factor (HGF) and IFNγ (Figure 7H).

Most of the effects of LBQ657 were dose‐dependent, except for HGF, IFNγ, and TGFβ which were effectively modified by both, low‐ and high‐dose LBQ657. Improved liver sinusoidal permeability by LBQ657 was evident from reduced CD31 expression (Figure 7E) and increased VEGF to the level of vehicle control (Figure 7H). This leads to speculation that LBQ657 when acting on the liver endothelium, may enhance the permeability of LSEC. As a result, blood‐derived Aβ is delivered more efficiently to the liver parenchyma.

Consistent with our data on CCl_4_‐treated 3xTg‐AD mice, the suppression of metalloproteinases MMP‐9 and −12 could also be achieved in BL/6‐CCl_4_ treated with LBQ657 (Figure 7I). Besides its Aβ‐degrading function, MMP‐9 activates the latent TGFβ, with subsequent HSC activation and collagen deposition.^[^ ^69^ ^]^ Moreover, increased expression of MMP‐9 is associated with EMT.^[^ ^46^ ^]^ The MMP‐9 inhibiting effect of LBQ657 has been previously observed in a model of TGFβ‐induced cardiac fibrosis,^[^ ^67^ ^]^ which, however, was mainly attributed to the inhibitory effect on the transient receptor potential melastatin‐like 7 (TRPM7) channel.

In summary, the data obtained on LBQ657 in the CCl_4_ model allowed to establish the inhibition of Aβ degradation as a feasible approach to promote the anti‐fibrotic effects of Aβ. The notion that increased systemic and intrahepatic Aβ levels in 3xTg‐AD mice and treatment of CCl_4_‐exposed WT mice with LBQ657 exert effects opposite to those observed in APP‐KO and 3D6‐immunized mice reinforces the essential role of Aβ in liver defense against fibrosis.

## 3 Conclusion

**Figure 8:**
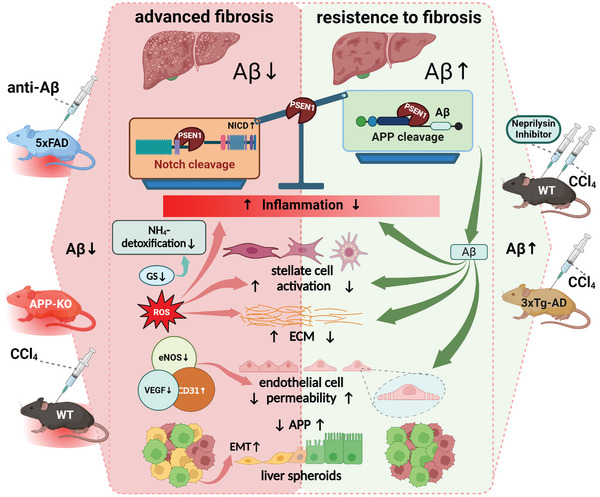
Schematic presentation of processes regulated by Aβ in the liver. APP‐ and NOTCH‐cleaving enzyme PSEN1 favors NOTCH processing in the situation of decreased hepatic levels of Aβ during fibrosis. Left panel: neutralization of Aβ by 3D6‐antibody treatment (anti‐Aβ) or induction of liver fibrosis in wild type (WT) mice by CCl_4_ or amyloid precursor protein (APP) knockout (APP‐KO) results in downregulation of Aβ in the liver leading to decreased ammonia detoxification by glutamine synthetase (GS), HSC activation, extracellular matrix (ECM) deposition, decreased liver endothelial cell (LSEC) permeability reflected by downregulation of eNOS and VEGF and an increased expression of CD31 in transgenic 5xFAD, CCl_4_‐treated wild type (WT) and APP‐KO mice; knockdown of APP in human liver spheroids induces epithelial‐mesenchymal transition (EMT) of hepatocytes. Right panel: high systemic and hepatic levels of Aβ in 3xTg‐AD mice and treatment of BL/6 mice with the neprilysin inhibitor sacubitrilat provide protection against liver fibrosis, normalizing the processes presented in the right panel. High hepatic Aβ level is accompanied by an overweight of PSEN1‐mediated APP cleavage over its NOTCH‐cleaving function. The figure was created with BioRender.com.

This study identifies soluble Aβ42 as a highly potent endogenous regulator of hepatic cell response to fibrogenic cues. Being delivered by blood as well as locally generated in the liver, Aβ counteracts fibrosis by reversing or suppressing a multitude of interconnected processes such as activation of NOTCH‐, TGFβ‐, TNFα/IL‐6/IL‐13 pathways subsequently leading to alleviated inflammation, ECM reorganization, activation of HSC, epithelial‐mesenchymal transition, and hepatocyte damage by oxidative stress (**Figure** **8**). By mediating autocrine and paracrine signals between HSC and LSEC, Aβ maintains liver sinusoidal permeability, thereby promoting nutrient supply to and detoxification function of hepatocytes. Acknowledging the recent developments in AD therapy aimed at reducing Aβ deposition in the brain, our results suggest that antibodies that do not deplete peripheral sources of Aβ will allow circumventing its deficiency in the liver, which could lead to the development of fibrosis over time. Another translational implication of Aβ function in the liver herein is that a high hepatic level of Aβ may provide powerful protection against liver fibrosis. The anti‐fibrotic features of Aβ explain why the inhibitor of neprilysin ameliorates liver fibrosis. Our data also suggest that during liver fibrosis cleavage activities of γ‐secretase strongly favor the activation of NOTCH rather than cleaving APP to produce Aβ. To this end, liver‐targeted gene therapy to enhance the expression of APP and BACE1 may help restore the balance in cleavage activities of γ‐secretase during fibrosis.

## 4 Experimental Section

### Animal Models and Treatments—Aβ Antibody Treatment of WT and 5xFAD Mice

Six‐month‐old female 5xFAD mice harboring five familial AD (FAD) mutations [APP K670N/M671L (Swedish) + I716V (Florida) + V717I (London) and PS1 M146L+ L286V]^[^ ^70^ ^]^ were bred heterozygous on a C57Bl/6J background. Female 5xFAD and wild type (WT, C57BL/6J) littermates were treated with weekly intraperitoneal injections of Aβ‐specific antibody 3D6 (IgG2a subtype, 20 mg kg^−1^) or an IgG2a isotype control for 6 weeks. For 8‐month immunization, 3–4‐month‐old 5xFAD animals were used. In an 8‐month immunization study, the applied dose was 12 mg kg^−1^ per injection for both antibodies. Animals were sacrificed by CO_2_ and isolated livers were stored at −80 °C until use.

### Animal Models and Treatments—APP‐KO Mice

APP‐KO mice were described previously^[^ ^71^ ^]^ and maintained on a C57BL/6 background. For assessment of fibrotic‐like changes in the liver, 28–37 weeks old male and female APP‐KO mice and their WT controls (C57BL/6) were euthanized under CO_2_ anesthesia. The livers were shock‐frozen and kept at −80 °C until further processing with immunohistochemistry, Western Blots, and Multiplex analyses.

### Carbon Tetrachloride‐Induced Fibrosis and LBQ657 Treatment

Carbon tetrachloride (CCl_4_, Sigma, Deisenhofen, Germany) was diluted in corn oil (Sigma). Female and male 3xTg‐AD mice harboring PS1_M146V_, APP_Swe_, and tau_P301L_ transgenes^[^ ^72^ ^]^ (Jackson Laboratories) and their WT controls (C57BL/6J) were injected intraperitoneally with 50 µL CCl_4_ in a final dose of 0,7 µL g^−1^ body weight twice a week (*n* = 12 per group). Control BL/6 animals were injected with the solvent (corn oil) only. Two groups of BL/6 mice (*n* = 8) received either 5 or 30 mg kg^−1^ body weight sacubitrilat (LBQ657, Hoelzel Diagnostika GmBH, Germany), injected daily over the entire period of 5‐week CCl_4_ treatment. All animals were euthanized under CO_2_ anesthesia. Blood was obtained by cardiac puncture and centrifuged after 30 min with 4.000 g at 4 °C for 10 min. Blood plasma and tissue samples were frozen at −80 °C until use.

### Cell Culture

Human SV40‐immortalized hepatic sinusoidal endothelial cells (hLSEC, Applied Biological Materials, Richmond, BC, Canada), immortalized human hepatic stellate cells (LX2), HepG2 (ATCC), mouse primary hepatocytes, human primary hepatic stellate cells (Innoprot), primary human liver sinusoidal endothelial cells (Innoprot) were cultivated as indicated in Supporting Information.

### Human Liver Spheroids

Cryopreserved primary human hepatocytes (PHH) (BioIVT) and primary human stellate cells (HSCs; Lonza) were co‐cultured in ultra‐low attachment plates (Sigma) at a ratio 4:1 as previously described.^[^ ^73^ ^]^ Spheroids were treated for 1 week with a mixture of oleic and palmitic acid (400 µm of each). For APP knock‐down experiments, cells were transfected with ON‐TARGETplus Human APP (351) siRNA (Dharmacon) at a final concentration of 50 nm. Thereafter the spheroids were processed for qPCR and immunofluorescence analyses as described in Supporting Information.

### Human Liver Samples

Pediatric liver tissues for mRNA expression analysis were histologically examined for patients with fibrosis (*n* = 9) and without fibrosis (*n* = 12) (for tissue characteristics see Table S2, Supporting Information). Tissue samples were obtained either during surgical resections or as snap‐frozen biopsy samples. Surgery or biopsies were done because of hepatoblastoma (*n* = 5), idiopathic hepatopathy (*n* = 5), congenital liver fibrosis (*n* = 5), or other diseases (*n* = 6), and as controls without fibrosis, only non‐affected tissue was used.

### Statistical Analyses

All normally distributed data were analyzed by One‐way ANOVA analysis with post hoc Bonferroni's multiple comparison test or two‐tailed Student's *t*‐tests for single comparisons. For non‐normally distributed data, appropriate non‐parametric analyses (Mann–Whitney or Kruskal–Wallis tests) were employed as specified in the respective Figure legends. Statistical analyses were performed using GraphPad Prism Software (GraphPad Software Inc, La Jolla, CA) and significance was defined as *p* < 0.05.

### Ethics Approval Statements

All animal experiments were approved by the local authorities of Animal Welfare in Tübingen (Regierungspräsidium Tübingen), Heidelberg (Regierungspräsidium Karlsruhe), and Halle (Landesverwaltungsamt Halle, approval number 42502‐2‐1369) conducted in accordance with the German federal law regarding the protection of animals and “Guide for the Care and Use of Laboratory Animals” (National Institutes of Health publication 8th Edition, 2011). Collection and use of human liver tissue samples and clinical data for this study were approved by the local Ethical Review Committee of the University of Regensburg (ethics statement 21‐2417‐101, University of Regensburg, Germany), with all patients providing informed consent for participation. All participant recruitment and informed consent processes were conducted in compliance with nationally accepted practice and in accordance with the World Medical Association Declaration of Helsinki 2018.

## Conflict of Interest

Eberhard Karls University of Tübingen in conjunction with the University Hospital of Tübingen has filed a patent covering Aβ‐enhancing strategies for the treatment of liver fibrosis where G.H.B, R.W., T.S.W, M.S., and L.D. are listed as inventors. V.M.L is a co‐founder, CEO, and shareholder of HepaPredict AB. All other authors declare no conflict of interest.

## Author Contributions

G.H.B. conceived the idea. G.H.B. and L.D. wrote the manuscript. L.D. and M.S. supervised the project. G.H.B., L.D., and U.S. designed the experiments. L.D., U.S., R.T., H.C., T.S.W., V.L., S.Y., I.R., M.V., J.‐U.R., V.R., M.B., V.W., and V.K. conducted experiments. G‐H.B., L.D., U.S., R.T., H.C., T.S.W., V.L., S.Y., I.R., M.V., T.S., K.K., and V.K. performed data analysis. U.C.M., R.W., and S.L.F. provided materials, discussed the results, and provided constructive comments on the manuscript. L.D., R.T., and M.S. contributed to the funding acquisition. V.L., R.G., S.L.F., U.C.M., and M.S. revised the manuscript for important intellectual content. All authors read and approved the manuscript.

## Supporting information

Supporting Information

## Data Availability

The data that support the findings of this study are available from the corresponding author upon reasonable request.
