# Supplementary material for: Consequences of Amyloid‐β Deficiency for the Liver
Source: Adv Sci (Weinh). 2024 Mar 2;11(18):2307734. doi: 10.1002/advs.202307734 (PMC11095235; doi:10.1002/advs.202307734)
Supplement: Supplementary file 1 — Supporting Information [file ADVS-11-2307734-s001.pdf]

ADVANCED SCIENCE
Open Access

Supporting Information

for *Adv. Sci.*, DOI 10.1002/advs.202307734

Consequences of Amyloid-β Deficiency for the Liver

Gayane Hrachia Buniatian, Ute Schwinghammer, Roman Tremmel, Holger Cynis, Thomas S. Weiss, Ralf Weiskirchen, Volker M. Lauschke, Sonia Youhanna, Isbaal Ramos, Maria Valcarcel, Torgom Seferyan, Jens-Ulrich Rahfeld, Vera Rieckmann, Kathrin Klein, Marine Buadze, Victoria Weber, Valentina Kolak, Rolf Gebhardt, Scott L. Friedman, Ulrike C. Müller, Matthias Schwab* and Lusine Danielyan**

Supporting Information

Consequences of Amyloid-β Deficiency for the Liver

Gayane Hrachia Buniatian, Ute Schwinghammer, Roman Tremmel, Holger Cynis, Thomas S. Weiss, Ralf Weiskirchen, Volker Lauschke, Sonia Youhanna, Isbaal Ramos, Maria Valcarcel, Torgom Seferyan, Jens-Ulrich Rahfeld, Vera Rieckmann, Kathrin Klein, Marine Buadze, Victoria Weber, Valentina Kolak, Rolf Gebhardt, Scott L. Friedman, Ulrike C. Müller, Matthias Schwab*, Lusine Danielyan**

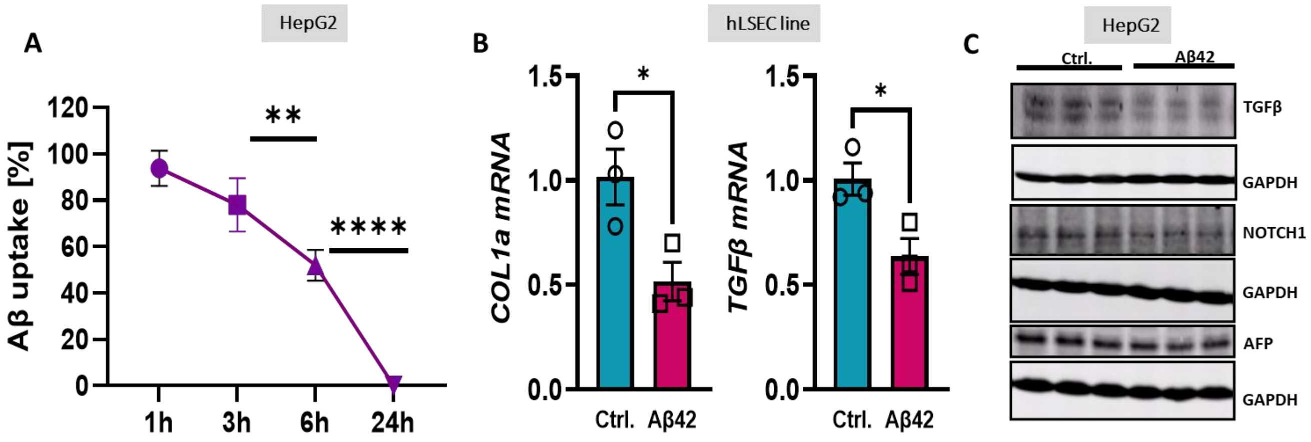

Figure S1. Aβ effects on HEPG2 and LSEC. A) Uptake of Aβ42 by HepG2 (n=4/group) reflected by reduced Aβ42 in culture supernatant in comparison to the initial amount (1000pg/ml) added to the culture at time point 0h; B) Collagen I and TGFβ mRNA expression in hLSEC line (n=3/group) incubated 24h with Aβ vs. control; C) Western Blot of TGFβ, Notch1 and AFP in HEPG2 incubated with Aβ42. The data are presented as means ± SEM. *p < 0.05, **p < 0.01, ****p < 0.001; 2-tailed Student's t-test (A and B).

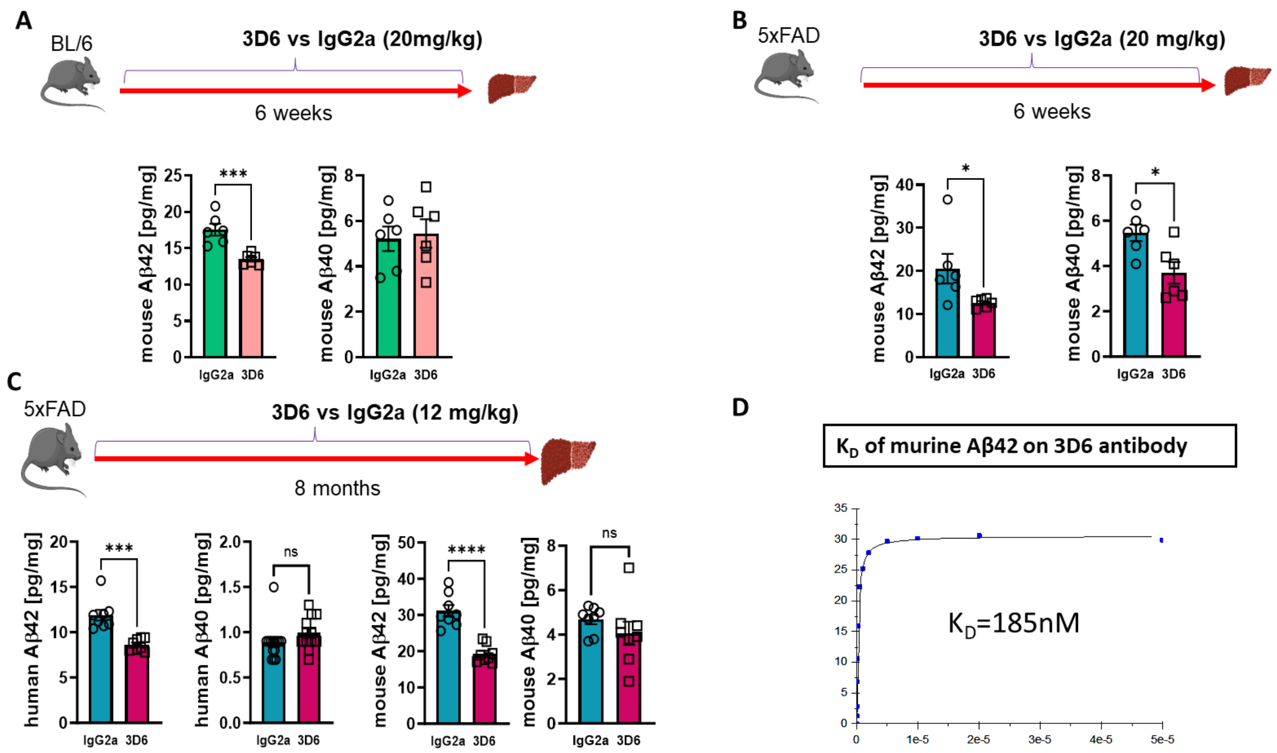

Figure S2. Quantification of Aβ in the liver of WT and transgenic mice. A) Mouse Aβ42 and Aβ 40 concentration in the liver of BL/6 mice after 6-week immunization with 3D6 vs. IgG2a control antibody (n=6/group); B) Mouse Aβ42 and Aβ40 concentration in the liver of 5xFAD mice after 6-week immunization with 3D6 vs. IgG2a control antibody (n=6); C) human and mouse Aβ42 and Aβ40 concentration in the liver of 5xFAD mice after 8-month immunization with 3D6 vs. IgG2a control antibodies; D) Surface Plasmon Resonance (SPR) spectroscopy analyzing the binding kinetics of mouse Aβ1-14 to 3D6_IgG2a. Steady-state-fit curve and K_D value. The data are presented as means ± SEM. *$p < 0.05$, ***$p < 0.005$, ****$p < 0.001$; 2-tailed Student's t test (A-C) and Mann-Whitney for Aβ40 analysis (in C)

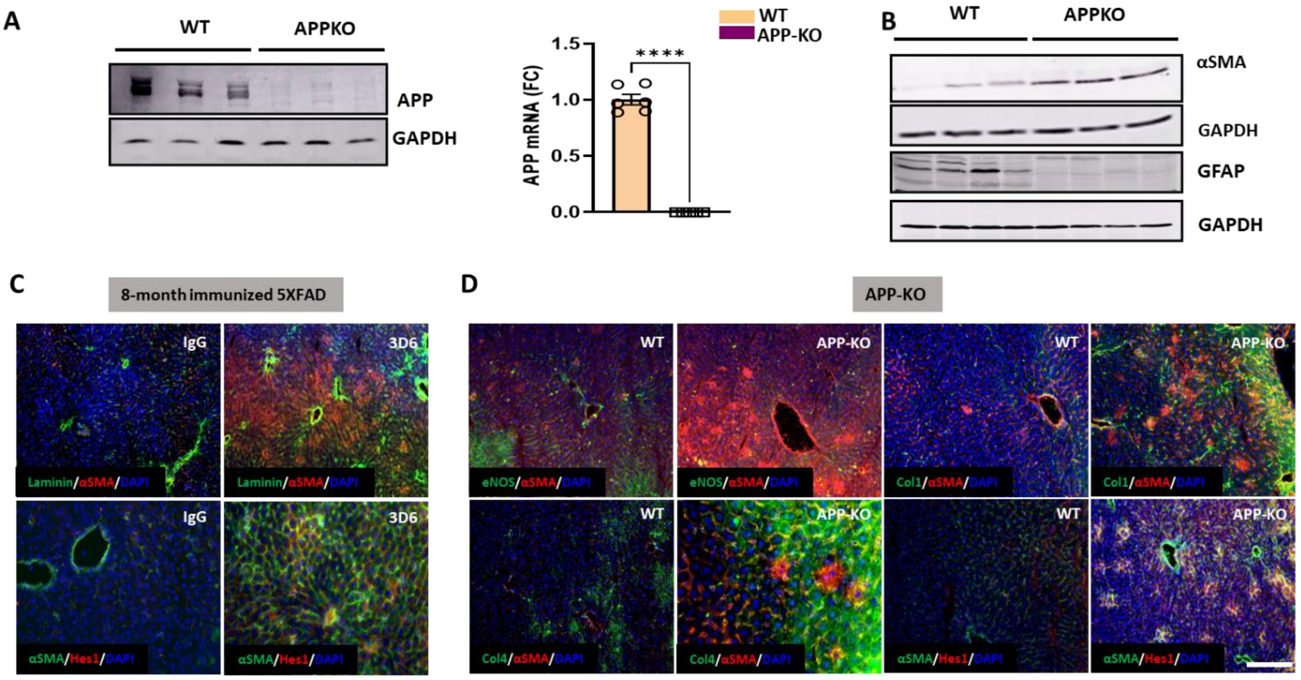

Figure S3. Fibrotic changes in the liver of APP-KO and immunized 5xFAD mice. A) Western Blot (n=3/group) and qPCR (n=6/group) showing APP expression in WT controls and its loss in the liver of APP-KO mice (n=3); B) Expression of αSMA and GFAP in the liver of WT and APP-KO mice (n=3); C) Representative images (out of n=4/group) of immunofluorescence analysis of Laminin1, Hes1 and αSMA in the liver of 5xFAD mice after 8-month immunization with 3D6 vs. control IgG2a antibody (n=8/group); D) Representative images of immunofluorescence analysis of eNOS, Collagen 4, Hes1 and αSMA in the liver of APP-KO and WT mice (n=4/group). The data are presented as means ± SEM. ****$p < 0.001$; Mann-Whitney test (A).

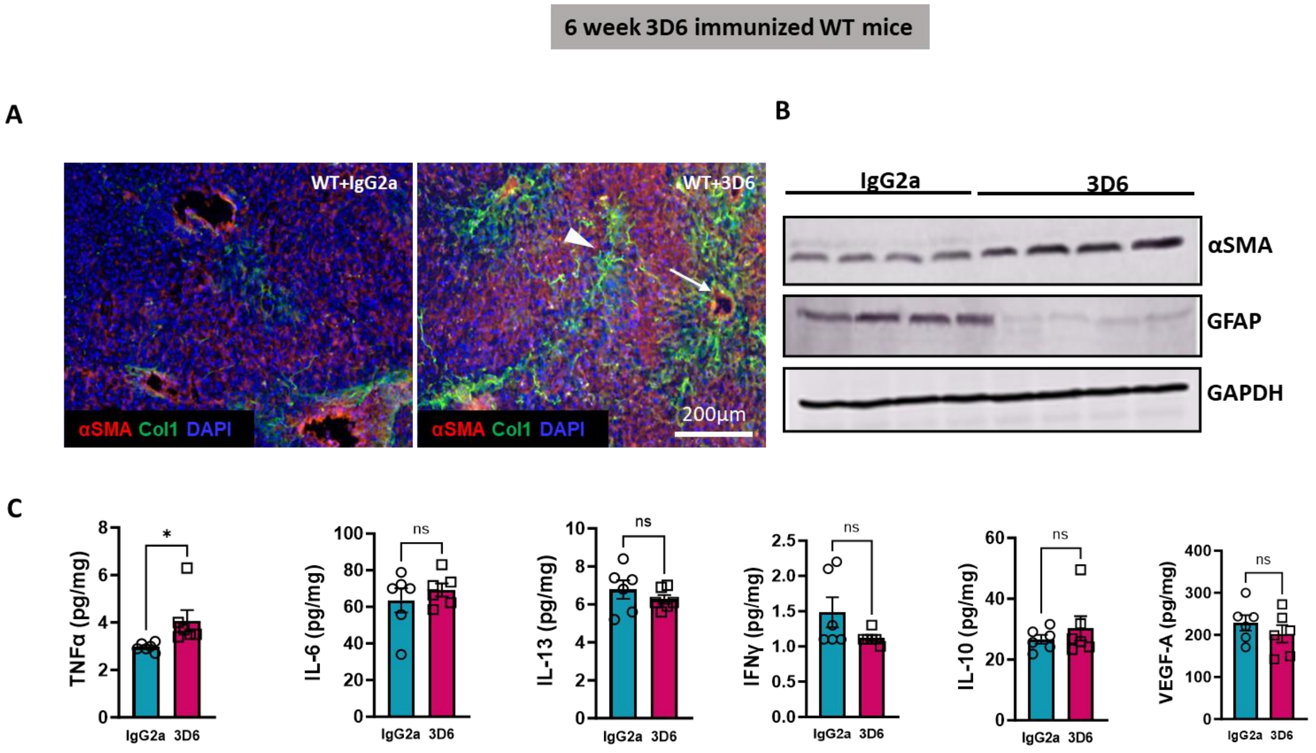

Figure S4. Liver fibrotic and inflammatory markers in 3D6 immunized WT mice. (A-B) expression of αSMA, collagen, and GFAP in the liver of WT (BL/6) mice 6 weeks after immunization using 3D6- vs. control IgG2a antibody (n=4/group); C) Multiplex analysis of TNFα, IL-6, IL-13, IFNγ, Il-10 and VEGF-A in the liver homogenates of WT mice (BL/6) after 6-week immunization with 3D6- vs. IgG2 (n=6/group); The data are presented as means ± SEM. $*p < 0.05$; 2-tailed Student's t-test for IL-6, IL-13, IL10 and VEGF-A (C) and Mann-Whitney test for TNFα and IFNγ (C).

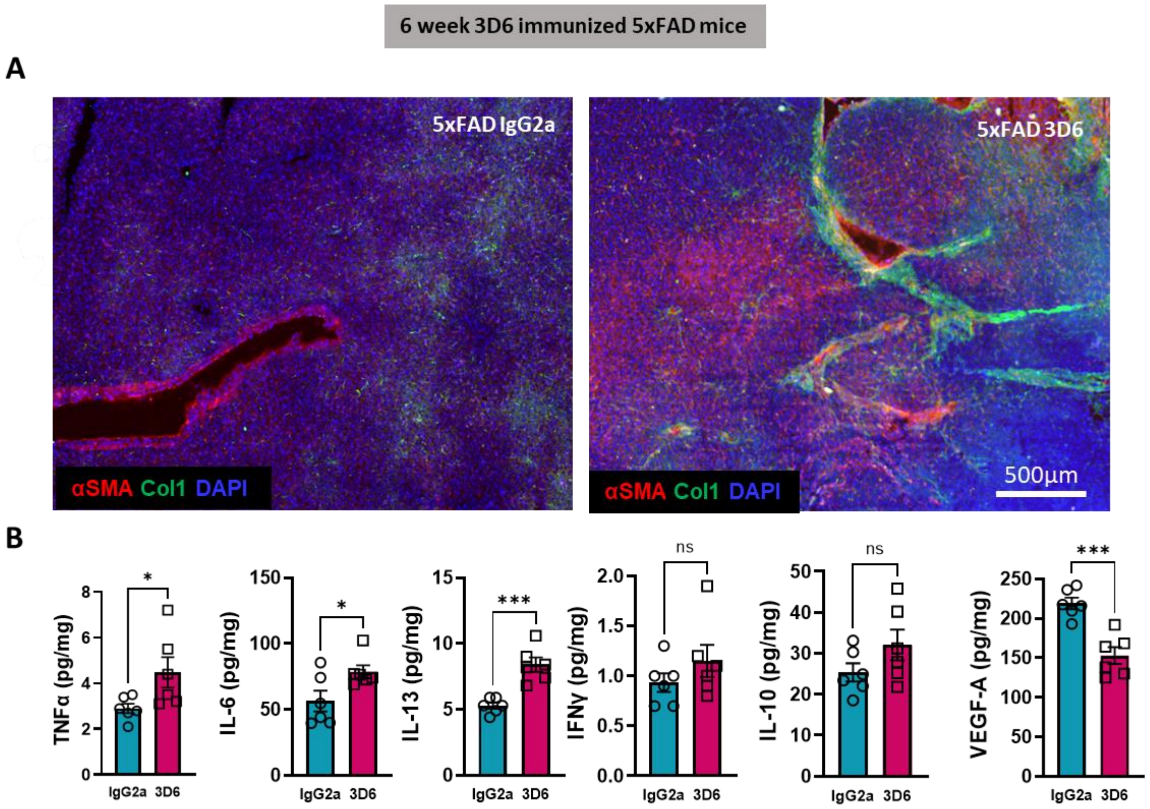

Figure S5. Liver fibrotic and inflammatory markers in 3D6 immunized 5XFAD mice. A) representative images of αSMA and collagen 1 expression in the liver of 5xFAD mice after 6-week immunization using 3D6- vs. control IgG2a antibody (n=4/group); B) Multiplex analysis of TNFα, IL-6, IL-13, IFNγ, IL-10 and VEGF-A in the liver homogenates of 5xFAD mice after 6-weeks immunization with 3D6- vs. IgG2 antibody (n=6/group); The data are presented as means ± SEM. *p < 0.05; 2-tailed Student's t test (B).

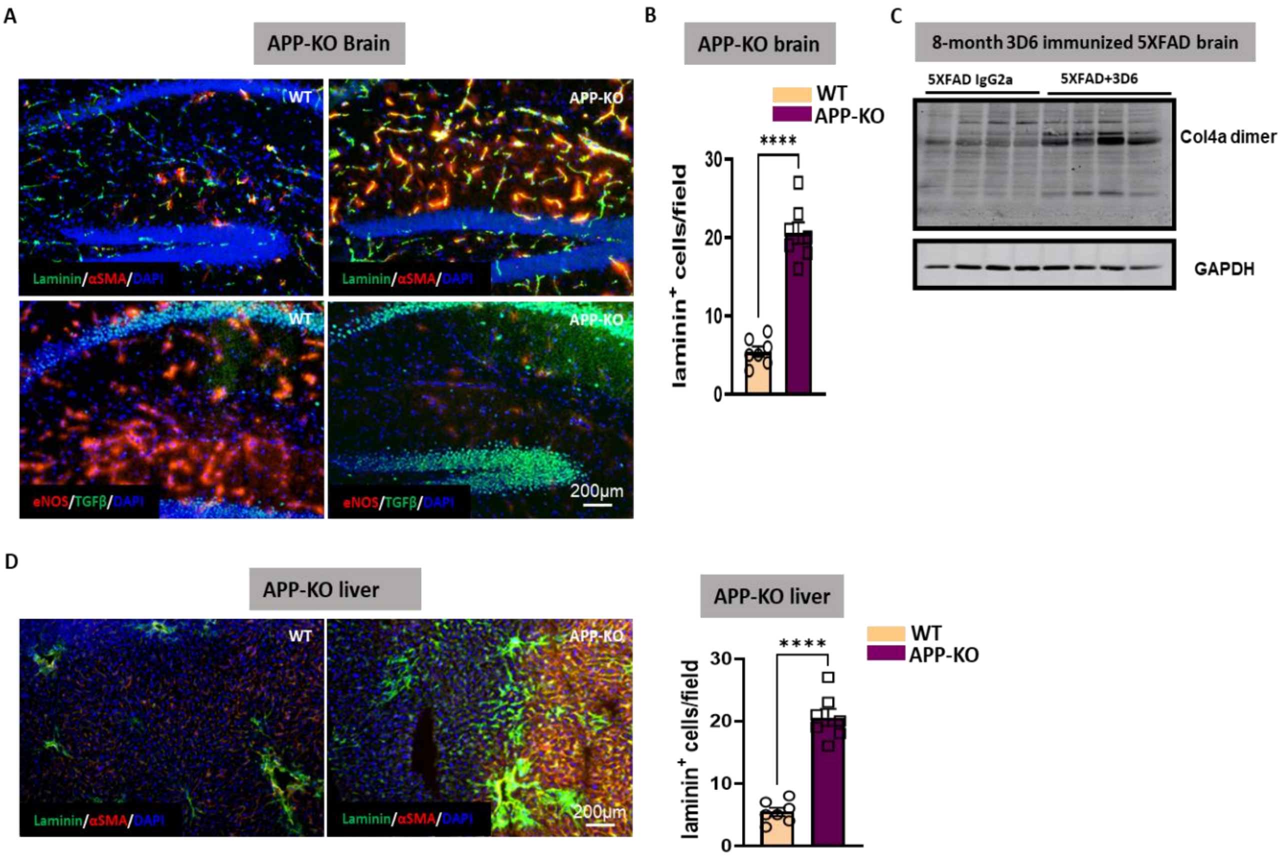

Figure S6. Laminin-1, αSMA, eNOS, and TGFβ in the brain and liver of APP-KO mice. A) representative images of laminin 1, αSMA, eNOS and TGFβ expression in the hippocampus of APP-KO vs. WT mice (n=4/group); B) Quantification of laminin1^{+}cells in the brain sections of APPKO vs. WT mice (n=3 mice/group, 7 sections/mouse); C) Western Blot of Collagen 4a in brain homogenates of 3D6 vs. IgG2 immunized 5XFAD mice after 9 months of immunization (n=4); D) representative images of Laminin/αSMA expression in the liver of APP-KO and WT mice and quantification of laminin-1^{+}cells in the liver sections of APPKO vs. WT mice (n=3 mice/group, 7 sections/mouse); The data are presented as means ± SEM. ***$p < 0.005$; 2-tailed Student's t-test (B, D).

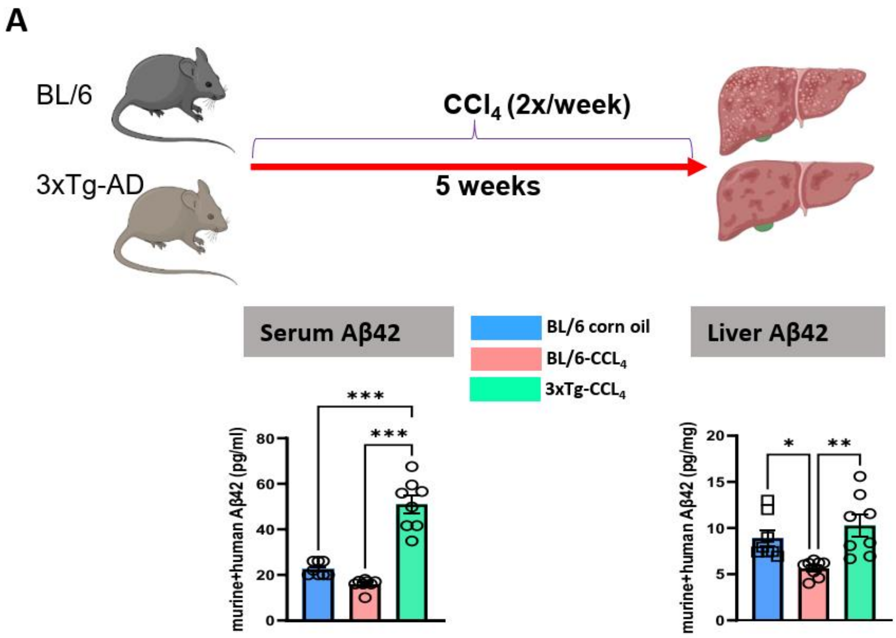

Figure S7. Quantification of Aβ42 in the liver of CCl_4-treated WT and 3xTg-AD mice. Aβ42 concentrations in the serum (n=7/group) and liver tissue (n=8/group) of mice after 5 weeks of treatment (twice per week) with CCl_4 (BL/6-CCl_4 and 3xTg-CCl_4) or corn oil (BL/6-Ctrl.). *$p < 0.05$, **$p < 0.01$, ***$p < 0.005$, ****$p < 0.001$; Kruskal-Wallis (for serum Aβ42) and 1-way ANOVA with Bonferroni's post hoc test (for liver Aβ42).

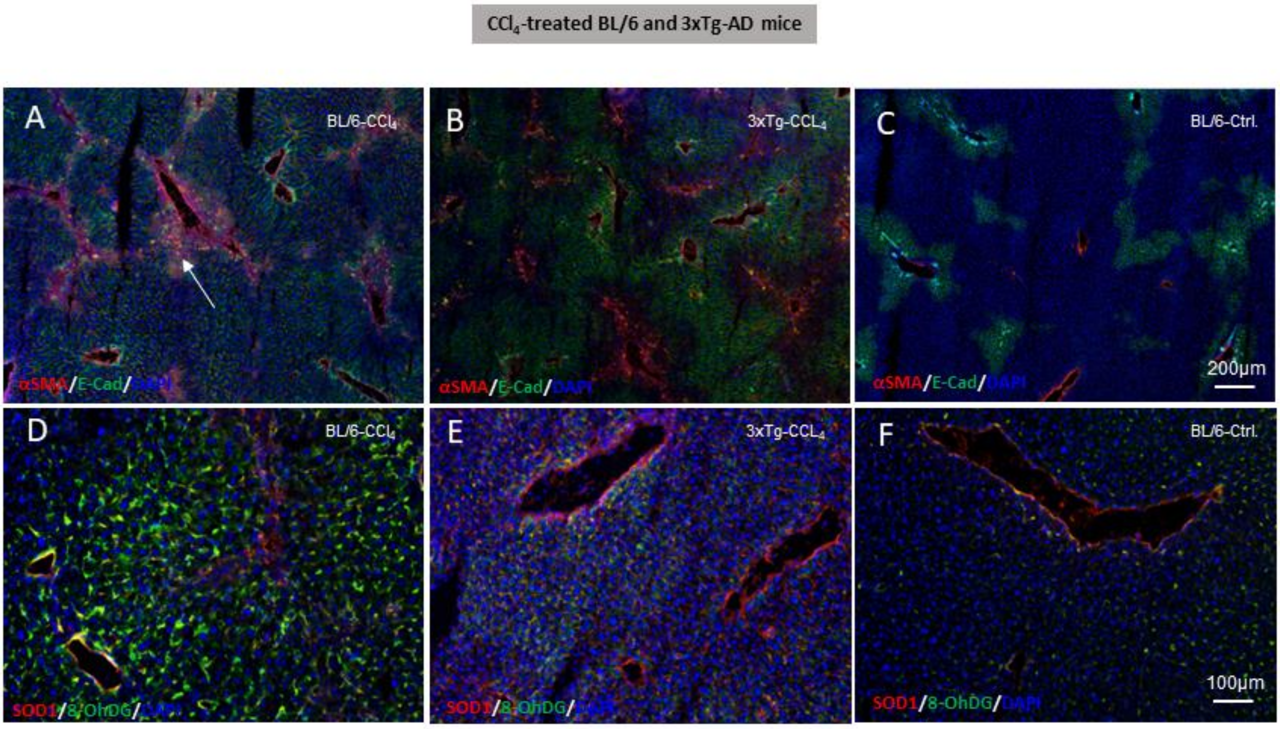

Figure S8. Aβ protects 3xTg-AD mice from CCl_4-induced oxidative stress and E-Cadherin reduction in pericentral hepatocytes. Liver sections of CCl_4- treated 3xTg-AD (3xTg-CCl_4) vs. BL/6 (BL/6-CCl_4) and corn oil-treated BL/6J controls (BL/6-Ctrl.) after 5 weeks of CCl_4 vs. Corn oil treatment; (A-C) Representative images of αSMA/E-Cadherin staining (n=4/group); (D-F) Immunofluorescence staining of liver sections for SOD1/8-OhDG, (n=4/group).

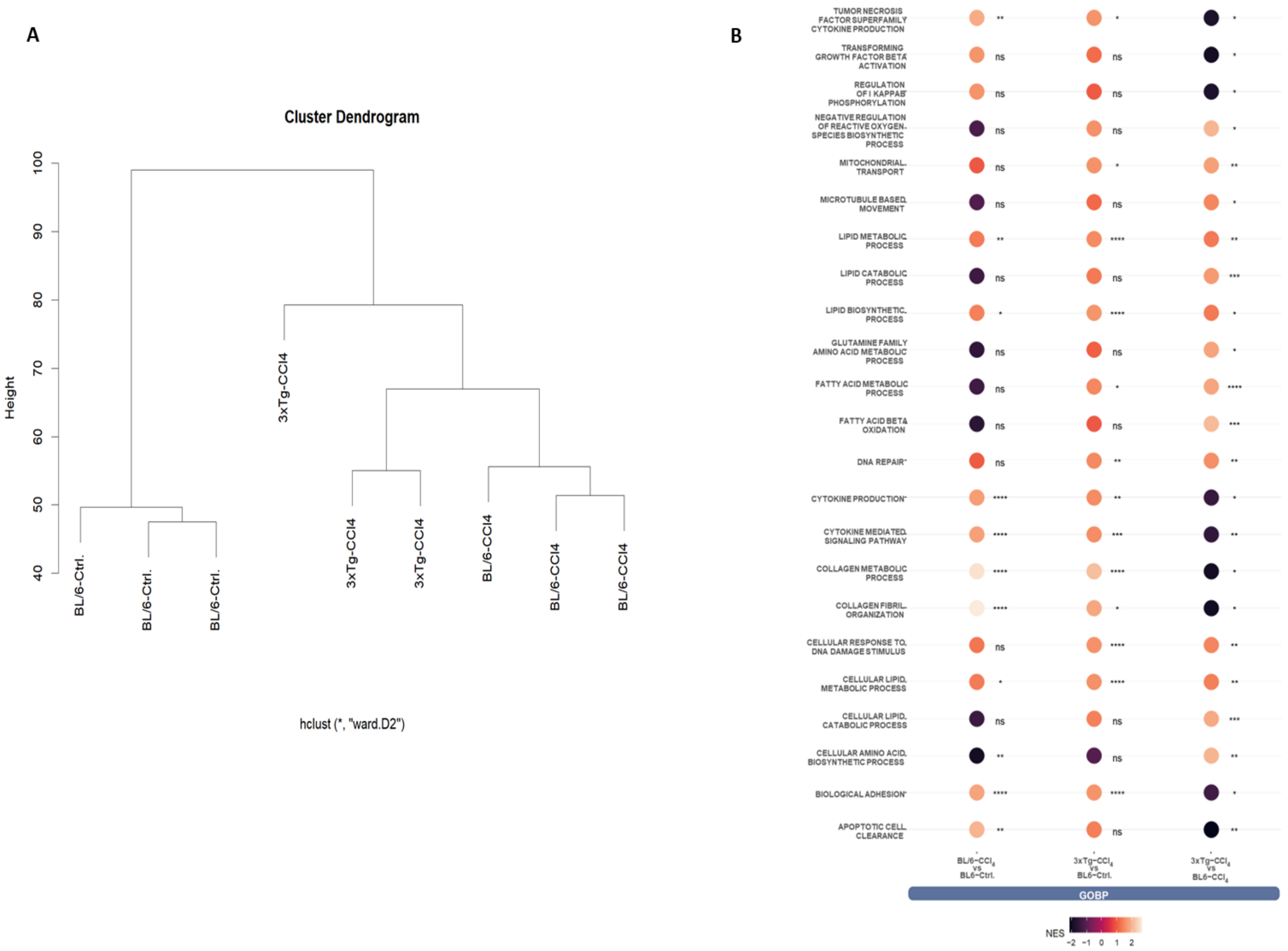

Figure S9. Transcriptome analysis of BL/6 and 3xTg-AD mouse livers. Liver samples from CCl_4-treated BL/6 and 3xTg and corn oil-treated BL/6 control mice were analyzed using Clariom S arrays. Results were filtered for FDR p-value <0.05 and absolute logFC >1.5. A) Enriched Gene Ontology (GO) biological process pathways. The color legend indicates the degree of normalized enrichment score (NES). Significance is reflected by *p*****=10-4, ***=10-3, **10-2, *=0.05, and ns=not significant; B) Hierarchical cluster analysis of the transcriptomic data. The cluster results from three mice of each group are plotted using a dendrogram.

Supplementary Methods

Antibodies and Peptides

Murine anti-Aβ(1-5)-specific antibody 3D6 (IgG2a) and IgG2a isotype control were recombinantly expressed in Freestyle 293-F cells (Thermo Fisher Scientific) essentially as described elsewhere [1]. Murine/Rat Aβ(1-14) for SPR analysis was purchased from peptides&elephants, Henningsdorf, Germany. Synthetic Aβ42 (MERCK) was dissolved in Tris-HCL according to the manufacturer's recommendation and kept frozen until use. For cell culture experiments thawed Aβ42 stock solution was rapidly added to the standard culture medium of the respective cell type (hepatocytes, hepatic stellate cells, or liver endothelial sinusoidal cells) and immediately used for the incubation with cells.

Cell culture

Human SV40-immortalized hepatic sinusoidal endothelial cells (hLSEC, Applied Biological Materials (Richmond, BC, Canada), immortalized human hepatic stellate cells (LX2) and HepG2 (ATCC) were grown in DMEM with high (4.5 g/l) glucose containing 5% or 10% fetal calf serum, 100 U/ml penicillin and 100 µg/ml streptomycin (Thermo Fisher Scientific, Darmstadt, Germany). Mouse primary hepatocytes were cultured in HepatoZyme Medium (Thermo Fisher) with 1 % penicillin/streptomycin and 2 mM L-Glutamine. For primary hepatocytes and hLSEC, collagen-precoated culture flasks were used. STR analyses of LSEC, LX2 and HepG2 lines and mycoplasma testing were performed prior to the experiments. Primary human adult stellate hepatic cells (hHSC) were obtained from Innoprot (Derio, Spain). Cells were cultured in endotoxin-free stellate medium supplemented with 2% fetal bovine serum (FBS), 100 units/ml penicillin, 100 µg/ml streptomycin, and 1% stellate growth factors (all tissue culture reagents being from Innoprot). Primary human LSEC (Innoprot) were cultured in collagen-precoated culture flasks with endotoxin-free endothelial medium supplemented with 5% fetal bovine serum (FBS), 100 units/ml penicillin, 100 µg/ml streptomycin and 1% endothelial growth factors (all tissue culture reagents being from Innoprot). Cultures were maintained at 37 °C in a humidified atmosphere with 5% CO_2.

Real-time qPCR of human primary LSEC

The αSMA mRNA expression in human primary LSEC was determined by quantitative real-time PCR using TaqMan probes. Total cellular RNA was collected from pelleted primary LSEC by TRI reagent (T9424, Sigma-Aldrich, St Louis, MO, USA). DNase treatment was performed as follows; 17µL of each RNA were mixed with 1µL of DNase TURBO (Cat. AM1907, Thermo Fisher Scientific, Waltham, MA, USA) in a final volume of 20µL. Samples were incubated at 37ºC for 30 minutes, followed by 5 minutes at room temperature. Then, samples were centrifuged, and the supernatants were transferred to clean tubes. Amount and purity were determined by Nanodrop (Thermo Fisher, Waltham, MA, USA). Then, reverse transcription was performed using 30 ng/µL of each RNA sample according to manufacturer's instructions (High Capacity cDNA RT kit including RNase inhibitor, Cat.No. 4368814, Thermo Fisher Scientific). Master Mix was mixed with 10µL of each RNA sample. Negative and non-template controls were included. A sample without reverse transcriptase was performed for each sample. Reverse transcription was conducted in the GeneAmp 9600 Thermocycler (ThermoFisher Scientific). qPCR was performed on Bio-Rad CFX thermocycler (Bio-Rad Laboratories Inc, Hercules, California, USA) in tetraplicate. Briefly, PCR mixes were prepared following manufacturer's instructions (Taqman Universal PCR Master Mix Cat.4304437. Thermo Fisher Scientific). Each mix contained 1µL of cDNA per replicate (αSMA or GAPDH) and the corresponding Taqman Probe (Hs00426835_g1 for αSMA and Hs02786624_g1 for GAPDH). No-template control and negative controls were included and GAPDH was used as a reference gene for endogenous control and for calculation purposes. For each sample, 4 replicate Cq values were obtained for each gene of interest and the reference gene. The mean Cq values were obtained and then, the ΔCq values (Cq gen – Cq housekeeping) were calculated.

qPCR and immunofluorescence analysis of human liver spheroids

Gene expression of PHH-HSCs co-culture spheroids was profiled by qPCR. Briefly, RNA was extracted using the Zymo quick RNA Micro kit (Zymo Research) kit followed by cDNA synthesis using SuperScript III Reverse Transcriptase (Invitrogen). qPCRs were performed using TaqMan probes for APP (Hs00169098) and Collagen 1a1 (Hs00164004) from ThermoFisher Scientific. Expression levels were analyzed using the ΔΔCt method with GAPDH as a reference gene. For immunofluorescence analysis PHH-HSCs co-culture spheroids were fixed with 4% paraformaldehyde overnight and then washed with PBS. Subsequently, spheroids

were kept in 30% sucrose-PBS at 4 °C until sinking. Spheroids were then transferred into molds with OCT in an isopropanol dry ice bath. Cryo-embedded microtissues were sectioned at 10 µm thickness on a CryoStar NX70 cryostat (Epredia). Sections were incubated with monoclonal primary antibodies for αSMA (1:200 mouse-αSMA; Abcam) and CYP3A4 (1:1000 rabbit-α-CYP3A4; Cypex), mounted using Prolong Gold Antifade mounting reagent with DAPI (Thermo Fisher) and imaged on a Zeiss LSM880 confocal microscope. For αSMA quantifications FIJI software has been used to normalize the signal for αSMA to DAPI. Colocalization analysis has been done in FIJI using the object-based overlap analysis.

Western blot analyses

Liver tissue was homogenized in ice-cold lysis buffer (300mM NaCl, 50 mM Tris, 2 mM $MgCl_2$, 0.5% NP40) containing the 'Complete protease inhibitor' (Roche, Rotkreuz, Switzerland). The total protein was determined by DC Protein assay (Bio-Rad). Proteins were fractionated by SDS/PAGE (10 or 12% acrylamide) and transferred onto PVDF membranes (EMD Millipore, Billerica, CA, USA). Membranes were blocked in 5% BSA (Albumin Fraction V, protease-free, Roth, Germany) in TBST for 1 h and were incubated at 4°C overnight with respective primary antibodies (see Table S1) diluted in 5% BSA. For visualization of antibody binding, membranes were incubated with Cy3/Cy5-conjugated antibodies for 1-3 h at RT. Protein bands were visualized using chemiluminescence or fluorescence detection systems (Bio-Rad, Hercules, CA). For imaging a VersaDocTM 4000 MP imaging system (Bio-Rad, Hercules, CA) was used. For Western Blot analyses LSEC and HepG2 were seeded onto culture flasks pre-coated with collagen (in case of LSEC) at a density $2x10^6$ cells/$150cm^2$ and incubated for 48 hours with or without 1,000 pg/ml synthetic Aβ42 dissolved in standard medium and harvested for Western Blot analysis.

Surface Plasmon Resonance spectroscopy

Binding kinetics of murine/rat Aβ(1-14) to antibody 3D6 was assessed using surface plasmon resonance (SPR) spectroscopy using a Biacore 3000 essentially as described elsewhere [1]. Briefly, goat anti-mouse IgG was immobilized on a CM5 sensor Chip (GE Healthcare) followed by capturing of antibody 3D6. Binding of murine/rat Aβ(1-14) to 3D6 was determined by applying 10nM – 50 µM murine/rat Aβ(1-14) to 3D6 in a multi cycle kinetic analysis followed by calculation of kinetic constants over all recorded sensograms using the 1:1 Langmuir binding model.

Quantification of Aβ peptides in cell culture supernatants, liver and brain homogenates

Cell culture supernatants were analyzed with High Sensitivity Human Amyloid β42 (EZHS42, MERCK, Darmstadt, Germany). Mouse livers from transgenic AD (5XFAD and 3xTg-AD) mice with their respective WT controls were analyzed by Luminex assay using MILLIPLEX MAP Mouse and Human Amyloid Beta Magnetic Bead Kit (MERCK).

Quantification of cytokines and growth factors

Liver tissue was homogenized in ice-cold lysis buffer (300mM NaCl, 50mM Tris, 2mM $MgCl_2$, 0.5% NP40) containing 'Complete protease inhibitor' (Roche, Rotkreuz, Switzerland). The total protein was determined by DC Protein assay (Bio-Rad). VEGF-A, TNFα, IL-10, IFNγ, IL-13, IL-6, OPN, TGFβ, HGF, MMP-12 and MMP-9 were analyzed by Luminex with Procarta Plex (ThermoFisher Scientific) and R&D Systems Custom Panel assay according to the manufacturer's protocols.

Immunofluorescence analyses

For immunofluorescence analyses mouse primary hLSEC line and mouse primary hepatocytes were plated in 6 cm Petri dishes (2.5 x 10^5 cells/dish) containing coverslips. Cells were incubated with Aβ42 (1000 pg/ml) dissolved in the respective culture medium for 48 h.

Human primary LSEC were seeded at 30.000 cells per well on 96-well plate and incubated in a complete medium overnight in humidified 5% CO_2 atmosphere. Next day, cells were treated with Aβ42 (1000 pg/ml) in a complete medium for 48h. Then, cells were stained for 15 min with 4-Amino-5-Methylamino-2',7'-Difluorofluorescein Diacetate (DAF-FM, Termo Fisher) for assessment of NO by fluorescence microscopy.

Liver and brain tissue sections (10µm thick) and cells grown on glass cover slips were fixed in –20°C cold methanol, washed twice in PBS, and incubated for 1 h at room temperature or overnight at 4°C with primary antibodies (Table S1). After washing twice in PBS, samples were incubated with a corresponding fluorochrome-linked secondary antibody in the dark for 1 h at room temperature and were washed two times for 10 minutes with PBS containing 0.1% Triton® X-100 (Sigma, Deisenhofen, Germany). The slices and cells were then covered with Vectashield mounting medium (Vector Laboratories Burlingame, Burlingame, CA,

USA) containing 4',6 diamidino-2-phenylindole (DAPI), dried and stored at -20°C. As for negative controls, samples were treated with secondary antibodies only.

Immunostained tissues and cells were evaluated by fluorescence microscopy, using an Olympus BX51 Microscope (Olympus Optical Co. Europe, Hamburg, Germany). Images were captured by the digital camera F-View II and processed with software CellSens® (Olympus).

Primary and secondary antibodies were applied in concentrations according to manufacturer’s information.

FITC-Dextran permeability assay

Human LSEC line was grown in collagen-coated Petri dishes at a density of 300,000 cells/dish for 24h, incubated for 24h with Aβ42 (3000pg/ml) dissolved in a culture medium. Cells were then incubated to FITC-Dextran-150kDa (5mg/ml, MERCK) for 1h, washed, and assessed by Cytosmart live imaging system. Dextran-positive cells were analyzed by Image J software.

Cryosections and Sirius Red staining

Cryosections of 20µm thickness were obtained with a (Cryotom CM1950, Leica) and collected on slides (R. Langenbrinck GmbH, Emmendingen, Germany). Sections were dried for approximately 1h and then incubated for a minimum of 24h in Bouin’s-Solution (Sigma, Deisenhofen, Germany). Slides were then incubated for 1h in Sirius Red Solution (Abcam) and underwent serial washing in 0.01N HCL and then in ultra-pure water. Sections were then dehydrated in an ethanol series of 50%, 70%, 90% for 2 minutes each and three times for 2 minutes in 100% ethanol. The slides were dehydrated twice in Xylol for 2 minutes and embedded in DPX (Termo Fisher) and covered with coverslips (R. Langenbrinck GmbH, Emmendingen, Germany). Sirius Red staining was evaluated with a PANNORAMIC Scanner (3DHISTECH) and processed with the software SlideViewer (3DHISTECH).

ALT, AST and AP ELISA

Blood plasma samples of immunized 5xFAD, and CCl_4-treated 3xTG-AD mice with their respective WT controls were assessed for Mouse Aspartate Aminotransferase (AST, Abcam), Mouse Alkaline Phosphatase (AP, LSBio) and Mouse Alanine Aminotransferase (ALT, ABclonal) by ELISA according to the manufacturer´s protocols.

qPCR (Fluidigm) of human LSEC and mouse liver tissue samples

Gene expression was measured in human LSEC line and mouse liver using qPCR. Total RNA was isolated as recently described [4]. qPCRs were performed using TaqMan probes for human genes ACTA2 (Hs00426835_g1), COL1A1 (Hs00164004_m1), LAMB1 (Hs01055960_m1), TGFb (Hs00998133_m1), and mouse genes App (Mm01344172_m1), Adam10 (Mm00545742_m1), Adam17 (Mm00456428_m1), Col4a2 (Mm01210125_m1), Hes1 (Mm01342805_m1), Psen1 (Mm00501184_m1), Notch1 (Mm00435249_m1), and a BioMark HD system with 48.48 chips (Fluidigm, South San Francisco, CA), including 14 pre-amplification cycles of cDNA samples. Expression levels were analyzed using the ΔΔCt method with ACTB (Hs01060665_g1) and GAPDH (Hs01922876_u1) or Gapdh (Mm99999915_g1) and Hprt (Mm03024075_m1) as housekeeping controls.

Taqman quantitative Real time PCRs (qRT-PCRs)

Total RNA from 3D6- and IgG2-treated (control) 5XFAD mice (n≥11/group) was isolated using RNeasy Mini Kit (Qiagen, Hilden, Germany). One µg of total RNA was reverse-transcribed using the Reverse-Transcription System (Promega, Madison, WI, USA). Transcript levels of Notch1, and Ywhaz were quantified using real-time PCR technology (LightCycler, Roche, Penzberg, Germany). Primer pairs were designed as reported earlier for mNotch1 [2] and mYwhaz [3]. PCR reaction products were verified by sequence analysis and PCR analysis was performed in triplicates.

Total RNA from human liver tissues (normal liver, n = 12, fibrosis, n=9; see also Table S2) was isolated using RNeasy kit including on-column genomic DNA digestion with RNase-free DNase Set (Qiagen, Hilden, Germany). 750ng of total RNA was reverse-transcribed using TaqMan Reverse Transcription reagents from Applied Biosystems (Life Technologies. Carlsbad, USA). For quantitative RT-PCR analysis, we used Fluidigm’s BioMark high-throughput quantitative (q) PCR chip platform (Fluidigm Corporation, San Francisco, CA, USA) with predesigned gene expression assays from ThermoFisher (Table S3) according to the manufacturer’s instructions [5]. The data was analyzed using the ΔΔCt method [6] and the expression values were normalized to the expression levels of the housekeeping genes (GUSB, HPRT1, TBP).

Transcriptome analysis of mouse liver tissue

Total RNA extraction from CCl4-treated BL/6, 3xTg-AD, and corn oil-treated BL/6 liver samples was conducted using Trizol reagent (Life) according to the manufacturer's guidelines. High-quality RNA samples with a RIN number >7.0 were employed. Clariom S mouse arrays (Thermo Fisher Scientific, Waltham, Massachusetts) were utilized for microarray analysis. R with the additional packages oligo [7] and limma [8] were employed to analyze DEGs in the livers of CCl4-treated BL/6, 3xTg-AD, and corn oil-treated BL/6 controls. Genes with a false discovery rate (FDR) parameter below 0.05 and an absolute fold change of ≥1,5 were designated as DEGs. These DEGs underwent subsequent enrichment analysis using KEGG, GO molecular functions, and biological process pathways.

Supplementary Tables

Table S1. List of antibodies for immunofluorescence analysis and Western Blots

**Antibody/host**	**Manufacturer**	**Cat. No.**
NICD, rabbit	Cell Signaling	4147
αSMA, mouse	Progen	61001
αSMA rabbit	Abcam	ab124964
GFAP/rabbit	DAKO	Z0334
CD31(PECAM), rabbit	Abcam	ab281583
VEGF, rabbit	MERCK	ABS82
HES1, rabbit	abcam	ab108937
NICD, rabbit	Cell Signaling	4147
eNOS, rabbit	Abcam	95254
eNOS, mouse	BD Transduction Laboratories™	610296
TGFβ rabbit	Abcam	ab92486
Laminin, rabbit	Abcam	ab11575
Collagen-1 rabbit	MERCK	AB765P
APP, rabbit	Abcam	ab32136
E-cadherin	Cell Signaling	3195
PCNA, mouse	MERCK	CBL407
GAPDH, mouse	MERCK	MAB374
GS, rabbit	MERCK	G2781
Col4, rabbit	ThermoFisher	PA1-28534
SOD1, mouse	Santa Cruz	sc-101523
8-OhDG, rabbit	Bioss	BS-1278R
AFP, rabbit	ThermoFisher	(PA5-21004)

Table S2. Patient demographics and laboratory parameters. The data is presented as median values with a range of values (in brackets). *ALT* alanine aminotransferase, *AST* aspartate aminotransferase. Mann-Whitney U Test for independent groups and Chi-square test were used to analyze gender, fibrosis, inflammation and steatosis. A value of $p < 0.05$ was considered significant. Human liver tissues for mRNA expression analysis were histologically examined in patients with or without fibrosis. [a] Fibrosis was defined as (F0) no fibrosis, (F1) zone 3 perisinusoidal/pericellular fibrosis; focally or extensively present, (F2) zone 3 perisinusoidal/ pericellular fibrosis with focal or extensive periportal fibrosis, (F3) zone 3 perisinusoidal/pericellular fibrosis and portal fibrosis with focal or extensive bridging fibrosis and (F4) cirrhosis. [b] Inflammation was scored as (0) no foci / 20 × field, (1) <2 foci / 20 × field, (2) 2–4 foci /20×field and (3) >4 foci / 20 × field. [c] Steatosis was scored as (0) <5% steatosis, (1) 5 to 33% steatosis,´(2) >33 to 66% steatosis and (3) >66% steatosis.

	Normal liver	Fibrosis	P value
Patients (m/f)	12 (9/3)	9 (3/6)	
Age [y]	11 (1.2-18)	9.5 (0.9-21)	
BMI [kg/m^2]	18.1 (13.8-27.7)	18,2 (12.9-19.2)	
Fibrosis [a]	0 (0-1)	3 (1-4)	0,005
Inflammation [b]	0 (0-1)	0 (0-1)	
Steatosis [c]	0 (0-1)	0 (0-1)	
ALT (U/l)	41 (16-140)	45 (22-234)	
AST (U/l)	45 (29-106)	34 (14-406)	
Bilirubin (mg/ml)	0.7 (0.2-3.8)	1.3 (0.3-26.9)	

Table S3. List of genes and assay numbers (Thermo Fisher) used for Fluidigm qRT-PCR. Housekeeping genes: GUSB, Glucuronidase, beta; HPRT1, Hypoxanthine phosphoribosyl transferase 1; TBP, TATA box–binding protein.

Gene	*Assay No.*
ADAM9	Hs00177638_m1
ADAM10	Hs00153853_m1
ADAM17	Hs01041915_m1
BACE2	Hs00273238 m1
JAG1	Hs01070032_m1
MMP2	Hs01548727_m1
NOTCH3	Hs00153510_m1
LRP1	Hs00233856_m1
UCHL1	Hs00985157_m1
GUSB	Hs00939627_m1
HPRT1	Hs02800695_m1
TBP	Hs00427620_m1

Supplementary References

[1] Gnoth K., Piechotta A., Kleinschmidt M., Konrath S., Schenk M., Taudte N., Ramsbeck D., Rieckmann V., Geissler S., Eichentopf R., Barendrecht S., Hartlage-Rübsamen M, Demuth H.U., Roßner S., Cynis H., Rahfeld J.U., Schilling S. 2020 Targeting isoaspartate-modified Aβ rescues behavioral deficits in transgenic mice with Alzheimer's disease-like pathology. Alzheimer's Research & Therapy 12(1):149.
[2] Robinson, S.C., K. Klobucar, C.C. Pierre, A. Ansari, S. Zhenilo, E. Prokhortchouk, and J.M. Daniel. 2017. Kaiso differentially regulates components of the Notch signaling pathway in intestinal cells. *Cell Commun Signal*. 15:24.
[3] Weiss, T.S., M. Lupke, S. Ibrahim, C. Buechler, J. Lorenz, P. Ruemmele, U. Hofmann, M. Melter, and R. Dayoub. 2017. Attenuated lipotoxicity and apoptosis is linked to exogenous and endogenous augmenter of liver regeneration by different pathways. *PLoS One*. 12:e0184282.
[4] Schwinghammer UA, Melkonyan MM, Hunanyan L, Tremmel R, Weiskirchen R, Borkham-Kamphorst E, Schaeffeler E, Seferyan T, Mikulits W, Yenkoyan K, Schwab M, Danielyan L: α2-Adrenergic Receptor in Liver Fibrosis: Implications for the Adrenoblocker Mesedin. Cells, 2020, 9:456.
[5] Spurgeon SL, Jones RC, Ramakrishnan R. High throughput gene expression measurement with real time PCR in a microfluidic dynamic array. PLoS One 2008, 3, e1662.
[6] Livak KJ, Schmittgen TD. Analysis of relative gene expression data using real-time quantitative PCR and the 2(-Delta Delta C(T)) Method. Methods 2001, 25, 402–408.
[7] Carvalho BS, Irizarry RA (2010). "A Framework for Oligonucleotide Microarray Preprocessing." Bioinformatics, 26(19), 2363-7. ISSN 1367-4803, doi:10.1093/bioinformatics/btq431.
[8] Ritchie ME, Phipson B, Wu D, Hu Y, Law CW, Shi W, Smyth GK (2015). "limma powers differential expression analyses for RNA-sequencing and microarray studies." Nucleic Acids Research, 43(7), e47. doi:10.1093/nar/gkv007.
